# Preclinical and Phase 1 Assessment of Antisense Oligonucleotide Bepirovirsen in Hepatitis B Virus–Transgenic Mice and Healthy Human Volunteers: Support for Clinical Dose Selection and Evaluation of Safety, Tolerability, and Pharmacokinetics of Single and Multiple Doses

**DOI:** 10.1002/cpdd.1154

**Published:** 2022-08-16

**Authors:** Kelong Han, Dickens Theodore, Gina McMullen, Eric Swayze, Michael McCaleb, Gaetan Billioud, Stefan Wieland, Steve Hood, Melanie Paff, C. Frank Bennett, T. Jesse Kwoh

**Affiliations:** ^1^ GSK Collegeville Pennsylvania USA; ^2^ GSK Durham North Carolina USA; ^3^ Ionis Pharmaceuticals Inc. Carlsbad California USA; ^4^ The Scripps Research Institute La Jolla California USA; ^5^ GSK Stevenage UK; ^6^ Present address: NIPD Genetics Biotech Ltd Nicosia Cyprus; ^7^ Present address: Department of Biomedicine, University Hospital Basel University of Basel Basel Switzerland

**Keywords:** 2′‐MOE antisense oligonucleotide, bepirovirsen, chronic hepatitis B, first‐in‐human, pharmacokinetics

## Abstract

Dose‐dependent reductions in hepatitis B virus (HBV) RNA, DNA, and viral proteins following bepirovirsen administration were observed in HepG2.2.15 cells. In HBV‐transgenic mice treated at 50 mg/kg/wk, hepatic HBV RNA and DNA were reduced by 90% and 99%, respectively. Subsequently, a phase 1 first‐in‐human study assessed pharmacokinetics and tolerability of single (75–450 mg) and multiple (150–450 mg on days 1, 4, 8, 11, 15, and 22) subcutaneous bepirovirsen doses in 96 healthy volunteers. Bepirovirsen at all dose levels was rapidly absorbed (maximum plasma concentration 3–8 hours after dosing), rapidly distributed to peripheral tissues, and slowly eliminated (median plasma terminal half‐life: 22.5–24.6 days across cohorts). Plasma exposure (dose‐proportional at 150–450 mg) and concentration‐time profiles were similar following the first and sixth doses, suggesting little to no plasma accumulation (steady state achieved by day 22). Renal elimination of full‐length bepirovirsen accounted for <2% of the total dose. Across the single and multiple dose cohorts, 197 treatment‐emergent adverse events were reported, with 99% and 65% classified as mild in severity and local injection site reactions, respectively. In conclusion, bepirovirsen showed an acceptable safety profile in humans with observed pharmacokinetics consistent with the chemical class, warranting further evaluation of bepirovirsen in chronic HBV infection.

It is estimated that 292 million people were living with chronic hepatitis B virus (HBV) infection in 2016.^1^ Chronic HBV, characterized by liver inflammation, increases the risk of liver cirrhosis and development of hepatocellular carcinoma leading to death.^2^, ^3^, ^4^

The goal of chronic HBV therapy is to improve quality of life and survival by preventing disease progression; effective therapy for chronic HBV must ensure a degree of virological suppression that induces normalization of serum alanine aminotransferase (ALT) levels and prevention of complications.^2^, ^4^ Nucleos(t)ide analogs are the recommended first‐line therapy for chronic HBV owing to their ability to effectively suppress HBV DNA levels and reduce the likelihood of disease complications.^2^, ^4^ However, hepatitis B surface antigen (HBsAg; a hallmark of chronic infection) clearance rarely occurs, and even long‐term therapy may not result in HBsAg loss/prevent relapse.^2^, ^5^ HBsAg is presumed to play a major role in viral persistence by repressing host immune responses; HBsAg suppression may allow reconstitution of the immune response.^4^, ^6^

Antisense oligonucleotides targeting regions of the HBV genome have efficiently reduced serum HBsAg and HBV DNA in preclinical studies, both alone and in combination with standard nucleoside therapy.^7^ Bepirovirsen (GSK3228836), previously ISIS 505358 or IONIS‐HBV_Rx_, is a 2′‐*O*‐methoxyethyl (2′‐MOE)‐modified antisense oligonucleotide in development for the treatment of chronic HBV infection.^8^ The bepirovirsen‐binding site is present in HBV messenger RNAs (mRNAs), including pregenomic RNA (pgRNA), and is highly conserved across HBV genotypes; as such, bepirovirsen would be expected to reduce levels of HBV mRNAs and pgRNA through an RNase H1 mechanism.^9^ Bepirovirsen was selected from a pool of HBV‐targeted antisense oligonucleotide candidates based on its potency in reducing HBsAg levels in both in vitro and in vivo HBV expression systems, and its safety and tolerability profile in mice and monkeys. No unexpected toxicities were observed in nonclinical studies of bepirovirsen conducted before the phase 1 first‐in‐human study. The plasma pharmacokinetics (PK) and tissue distribution of bepirovirsen were investigated in the general toxicology studies, and the results were consistent with those observed for other 2′‐MOE antisense oligonucleotides.^10^

This manuscript reports findings of in vitro and in vivo studies that informed bepirovirsen dose selection for studies in humans. We also present data from the phase 1 first‐in‐human study for bepirovirsen (study 213725; ISIS 50538‐CS1), which evaluated the PK, safety, and tolerability of single and multiple doses of bepirovirsen in healthy volunteers.

## Methods

### Preclinical Studies

#### In Vitro Cell Culture Dose Response

HepG2.2.15 cells^11^ were plated in a 96‐well plate at 28 000 cells per well and transfected with antisense oligonucleotides, entecavir, and tenofovir disoproxil fumarate using Lipofectamine 2000 (Life Technologies, Carlsbad, California). The antisense oligonucleotides used in these studies are shown in Table S1. Cell culture medium was replaced 2 days after transfection. After a treatment period of ≈16 hours, RNA and DNA were isolated using RNeasy 96 Kit (Qiagen, Hilden, Germany; catalog no. 74182) and DNeasy 96 Blood & Tissue Kit (Qiagen; catalog no. 69581). HBV mRNA and DNA levels were measured by a quantitative real‐time polymerase chain reaction system (Life Technologies) using primer probe sets 3370 and 3371 (Integrated DNA Technologies, Coralville, Iowa), which detect pgRNA, S1, and S2. HBV mRNA levels were adjusted according to total RNA content, as measured by RiboGreen (Life Technologies) and HBV DNA levels according to total DNA content, as measured by PicoGreen (Life Technologies). Secreted HBsAg and hepatitis B e antigen (HBeAg) were quantified by enzyme‐linked immunosorbent assay (ELISA) assays specific for HBsAg (Abazyme LLC, Cambridge, Massachusetts) and HBeAg (International Immuno‐Diagnostics, Foster City, California).

#### In Vivo Dose Response

Male HBV transgenic mice (Tg[HBV 1.3 genome]Chi32 against C57BL/6 background^12^) aged 7–12 weeks and weight 18–25 g were obtained from Scripps Research (La Jolla, California) and housed in shoebox cages with cob bedding with ad lib access to food (Teclad Laboratory Diet, Harlan, Indianapolis, Indiana) and water with a 12‐hour light/dark cycle and daily health check. All experiments involving mice were performed at Scripps Research following the National Institutes of Health Guide for the Care and Use of Laboratory Animals and in vivaria accredited by the Association for Assessment and Accreditation of Laboratory Animal Care.

Mice were prebled to ensure sufficient serum HBeAg levels using the qualitative HBeAg ELISA from International Immuno‐Diagnostics, then injected subcutaneously with vehicle (saline) or bepirovirsen at various doses twice weekly for week 1 and once weekly for weeks 2–4, with and without cotherapy with entecavir administered via oral gavage. Mice were terminated 3 days after the last dose.

##### Hepatic HBV RNA and DNA

Liver tissues were harvested, and HBV RNA and HBV DNA were isolated from mouse liver using the RNeasy 96 Kit and the DNeasy 96 Blood & Tissue Kit (Qiagen), respectively, per manufacturer's instructions. Real‐time polymerase chain reaction was performed with the StepOne System (Life Technologies). The primer and probe sequences for analysis of HBV expression were designed using Primer Express Software (PE Applied Biosystems, Waltham, Massachusetts) to detect full‐length, pre‐S1, and pre‐S2 HBV mRNAs (forward CCAAACCTTCGGACGGAAA; reverse TGAGGCCCACTCCCATAGG; probe CCCATCATCCTGGGCTTTCGGAAAATX). The amount of hepatic HBV DNA was normalized to the amount of total DNA determined by PicoGreen; serum HBV DNA was normalized to amount of DNA in a pool of saline day 0 samples. The amount of each HBV mRNA was normalized to amount of total RNA determined by RiboGreen.

##### Serum HBsAg and HBeAg

Serum samples were collected by retro‐orbital puncture with nonheparinized blood collection tubes. HBsAg and HBeAg levels were measured using the HBsAg One ELISA (lower limit of quantitation [LLOQ], 0.4 ng/mL) and HBeAg ELISA (LLOQ, 1.5 PEI U/mL) (International Immuno‐Diagnostics) at dilutions of 1:5 to 1:2000 in phosphate buffered saline with 10% fetal bovine serum per manufacturer's instructions. Reductions from baseline were calculated for individual mice.

##### Histology

Liver tissue samples were collected at necropsy, fixed in formalin, and embedded in paraffin. Tissue was stained for hepatitis B core antigen as detailed in the Supplemental Methods.

### Phase 1: Clinical Study

#### Study Design

This phase 1, blinded, randomized, placebo‐controlled, dose‐escalation study in healthy volunteers was conducted at a single center (INC Research, Toronto, Ontario, Canada). The first participant was enrolled on November 12, 2013, and the last participant visit was on August 25, 2014. The protocol was reviewed and approved by an independent review board (Institutional Review Board Services, Aurora, Ontario, Canada) before study start. Written informed consent was obtained from all participants before study participation. The study was conducted in accordance with the International Council on Harmonization Guideline for Good Clinical Practice and the original principles embodied by the Declaration of Helsinki.

Four sequential 4‐participant single‐dose cohorts (n = 4 for each cohort, alternative to 4 and 4) were randomized to bepirovirsen or placebo in a 3:1 ratio. Placebo was 0.9% sterile sodium chloride provided by the study center. Single doses of bepirovirsen were administered at dose levels of 75 mg (cohort A), 150 mg (cohort B), 300 mg (cohort C), or 450 mg (cohort D). Participants were followed for 30 days after treatment.

The multiple‐dose portion of the study commenced after the participants in the 450‐mg single‐dose cohort had reached day 4 and had demonstrated an acceptable safety profile. Three sequential 4‐participant multiple‐dose cohorts were randomly assigned to bepirovirsen or placebo in a 3:1 ratio. Participants received 6 doses of 150 mg (cohort M1), 300 mg (cohort M2), or 450 mg (cohort M3) over 3 weeks (dosing on days 1, 4, 8, 11, 15, and 22) and were followed for 13 weeks after treatment. Initiation of the next dose/treatment level occurred after the site investigator and the medical monitor jointly agreed that safety data (day 4 for single‐dose cohort and day 29 for multiple‐dose cohort) supported escalation.

Participants were assigned to treatment according to a randomization schedule generated before the study by the study sponsor and delivered to the unblinded pharmacist at each study center. The pharmacy staff (or qualified designees) preparing the study treatment and monitoring the pharmacy records were unblinded throughout the study. Everyone else directly involved in the conduct of the study was blinded to treatment assignment.

Bepirovirsen or placebo was administered by subcutaneous (SC) injection into the upper arm, thigh, or abdomen. For dose levels ≥300 mg (cohorts C, D, M2, and M3), the dose was split into 2 equal injections and administered at 2 separate anatomic sites. Follow‐up assessments were conducted on days 2, 4, 8, 15, and 30 in the single‐dose cohorts, and days 23, 29, 36, 50, 71, 92, and 113 in the multiple‐dose cohorts. Stopping criteria were based on changes in liver chemistry, renal function, or platelet count (Supplemental Methods). If any of the protocol‐defined stopping criteria were met, the participant was permanently discontinued from bepirovirsen treatment.

#### Participants

Participants were men or nonpregnant and nonlactating women, 18–65 years of age, with a body mass index (BMI) ≤32.0 kg/m^2^. Participants were required to be healthy with no clinically significant abnormalities in medical history or screening laboratory values and no active infection requiring systemic antiviral or antimicrobial therapy not completed before day 1. Full eligibility criteria are detailed in the Supplemental Methods.

#### Assessments

The primary end point of the study was assessment of safety and tolerability of single and multiple doses of bepirovirsen. The secondary end point was assessment of plasma PK. For measurement of bepirovirsen plasma PK parameters, blood was collected in the single‐dose cohorts at day 1 (before dosing and 0.5, 1, 1.5, 2, 3, 4, 6, 8, and 12 hours after administration), day 2 (24 hours after administration), day 4 (72 hours after administration), and days 8 and 15 (any time). In the multiple‐dose cohorts, blood was collected at day 1 (before dosing and 0.5, 1, 1.5, 2, 3, 4, 6, 8, and 12 hours after administration); day 2 (24 hours after day 1 administration); day 4 (before dosing [72 hours after day 1 administration]); day 5 (24 hours after day 4 administration); days 8, 11, and 15 (before dosing); day 22 (before dosing and 0.5, 1, 1.5, 2, 3, 4, 6, 8, and 12 hours after administration); day 23 (24 hours after day 22 administration); and days 29, 36, 50, 71, 92, and 113 (any time).

Urine was collected from 0 to 24 hours after dosing on day 1 (single‐ and multiple‐dose cohorts) and day 22 (multiple‐dose cohorts only) to measure urinary excretion of bepirovirsen.

Human plasma and urine samples were analyzed for concentrations of bepirovirsen at Tandem Laboratories Inc. (San Diego, California; site has since closed) using a validated hybridization ELISA method for plasma (range, 1–100 ng/mL; LLOQ, 1 ng/mL) and a qualified ELISA method for urine (range, 2–100 ng/mL; LLOQ, 2 ng/mL), based on published methodology.^13^, ^14^

The following PK parameters were assessed: maximum plasma concentration (C_max_), minimum plasma concentration (C_min_), time to C_max_ (t_max_), area under the plasma concentration–time curve (AUC) from 0 to 24 hours, AUC from 0 to 168 hours, AUC from 0 to 336 hours in single‐dose cohorts only, apparent plasma clearance, terminal plasma elimination half‐life for multiple‐dose cohorts only, urine concentration, amount excreted in urine up to 24 hours, percentage of dose excreted in urine, and renal clearance. Plasma and urine PK parameters were calculated following the first dose (all cohorts) and the last (sixth) dose (multiple‐dose cohorts only).

Safety and tolerability assessments included documentation of adverse events (AEs), laboratory parameters, 12‐lead electrocardiogram, and vital signs. Treatment‐emergent AEs were defined as events that occurred after initiation of study treatment (bepirovirsen or placebo) and before the end of the follow‐up period. AEs were coded according to the Medical Dictionary for Regulatory Activities version 16.1.

#### Statistical Analyses

The sample size was based on prior experience with other antisense oligonucleotides to ensure an adequate initial assessment of safety, tolerability, and PK while minimizing the number of participants exposed to bepirovirsen.

Noncompartmental plasma PK analysis of bepirovirsen was carried out on each individual participant's data set, where full PK concentration‐time profiles were analyzed using WinNonLin Professional version 5.3 or higher (Pharsight Corp., Mountain View, California). Plasma concentrations in 2 samples, 1 from cohort C and 1 from cohort M3, were considered as outliers (0 or near 0 concentrations partway through the plasma concentration–time curve) and excluded from analyses.

As there was only 1 placebo‐treated participant within each cohort, the placebo participants were pooled and analyzed as separate single‐dose and multiple‐dose placebo groups. The safety population included all participants who were randomized and received at least 1 dose of bepirovirsen; this population was used for all demographic, disposition, and safety analyses. The PK population included all participants who were randomized, received at least 1 dose of bepirovirsen and had any postdose plasma PK data. There were no changes in the conduct of the study after participant enrollment had commenced.

## Results

### Preclinical Studies

#### Dose‐Dependent Antiviral Activity of Bepirovirsen In Vitro

Bepirovirsen reduced levels of all HBV RNA transcripts in HBV‐expressing HepG2.2.15 cell cultures in a potent, sequence‐specific, and dose‐dependent manner (Figure S1A), *P* < .0001 by 2‐way analysis of variance with Tukeys's posttest. The half maximal effective concentration for reduction of HBV RNA was ≈31 nM. Similar potency was observed in different sets of experiments using multiple primer‐probe sets able to measure the 3.5‐kb HBV RNA transcript, the 2.1‐, 2.5‐, and 3.5‐kb transcripts, and all other HBV transcripts (Figure S2). In contrast, a non‐HBV‐targeting, control antisense oligonucleotide, ISIS 129700, showed no reduction of HBV RNAs at any concentration tested (Figure S1A). The bepirovirsen‐mediated reduction of HBV RNA also resulted in a potent reduction in the level of viral DNA and a dose‐responsive reduction in HBsAg and HBeAg (Figure S1B–D).

In cells treated with bepirovirsen, entecavir or tenofovir disoproxil fumarate, all agents gave nearly 50% inhibition of HBV DNA when administered alone. The addition of bepirovirsen to entecavir or tenofovir disoproxil fumarate increased the efficacy of HBV DNA reduction. Likewise, the addition of entecavir or tenofovir disoproxil fumarate to bepirovirsen resulted in increased efficacy (Figure S3).

#### In Vivo Dose‐Dependent Antiviral Activity of Bepirovirsen

HBV‐transgenic mice^12^ were used to demonstrate that bepirovirsen could reduce hepatic RNA transcripts, subsequent production of HBV DNA, and associated HBV serum antigens. To allow a robust assessment of changes in HBsAg levels following treatment with HBV antisense oligonucleotide, a set of HBV‐transgenic mice were prescreened for high serum HBeAg levels and then allocated into treatment groups based on HBsAg antigen level. Serum HBV DNA, HBsAg, and HBeAg were measured at baseline and then weekly during the 4‐week testing period.

**Figure 1:**
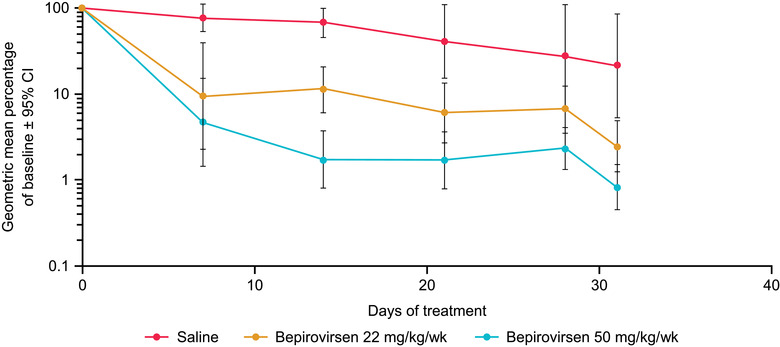
Dose‐dependent reduction in HBsAg in HBV transgenic mice treated with saline or bepirovirsen for 4 weeks. N = 8–12 mice per group. Data are expressed as geometric mean ± 95% CI. CI, confidence interval; HBsAg, hepatitis B surface antigen; HBV, hepatitis B virus.

Bepirovirsen dose‐dependently reduced hepatic expression of HBV RNA in HBV transgenic mice, with a maximal reduction >90% achieved following a dose of 50 mg/kg/week (Figure S4A). Hepatic levels of HBV DNA were similarly reduced (Figure S4B). Importantly, hepatic levels of HBV RNA and DNA were reduced *>*1 log in all mice treated. Consistent with the observed hepatic reduction of HBV RNA and DNA, serum HBV DNA was reduced *>*95% during treatment with bepirovirsen (Figure S5), with reductions observed from 7 days after dosing. Likewise, serum HBsAg levels were dose‐dependently reduced from 7 days after initial bepirovirsen administration, and low serum levels of antigen were maintained during the 4‐week testing period (Figure 1 and Figure S6). Maximal reduction of serum HBsAg was ≈99% (2 logs). Serum levels of HBeAg were also significantly reduced within 1 week, and low levels were maintained during the 4‐week testing period (Figure S7).

To qualitatively assess the ability of bepirovirsen to reduce hepatic levels of hepatitis B core antigen in HBV‐transgenic mice, representative samples of liver were stained for hepatitis B core antigen. As shown in representative liver sections (Figure S8), bepirovirsen reduced levels of all cytoplasmic hepatitis B core antigen, a marker of active HBV gene expression and replication.^12^

Treatment with entecavir alone did not affect HBV RNA levels, but reduced liver HBV DNA levels. This reduction was either unchanged or enhanced by coadministration with bepirovirsen. To assess the ability of bepirovirsen combined with nucleoside antiviral treatment, a separate dose‐response experiment was performed to measure the response to bepirovirsen on liver HBV RNA and DNA in the presence or absence of concomitant treatment with entecavir. Bepirovirsen exhibited dose‐dependent reductions in both HBV RNA and DNA (Figure S9A and B), with reductions similar to those observed previously, and a 99% reduction in HBV liver DNA at the highest dose (Figure S9B).

#### Dose Selection for Phase 1 Clinical Study

Preclinical in vivo data was used to predict the human dose. The preclinical transgenic mouse model showed that the 90% effective dose for HBsAg reduction was ≈20 mg/kg, which corresponds to a plasma PK exposure equivalent dose of 280 mg (4 mg/kg) of bepirovirsen in humans.^15^, ^16^, ^17^ Additional support for dose selection is detailed in the Supplemental Methods.

### Phase 1: Clinical Study

#### Participants

Overall, 96 healthy volunteers were screened, among whom 28 were randomly assigned into the study (single‐dose cohorts, n = 16; multiple‐dose cohorts, n = 12) (Figure S10). All randomly assigned participants received all scheduled doses and completed the posttreatment follow‐up according to the study schedule.

Baseline demographics and clinical characteristics were balanced across cohorts (Table S2). Over the full study population, there were 22 male and 6 female participants. In the single‐dose cohorts, the mean (standard deviation) age was 47.9 (11.7) years, BMI was 26.0 (2.4) kg/m^2^, and weight was 80.1 (11.2) kg at baseline. In the multiple‐dose cohorts, the mean (standard deviation) age was 51.1 (9.8) years, BMI was 27.1 (2.3), and weight was 80.2 (10.4) kg at baseline.

#### Plasma PK

**Figure 2:**
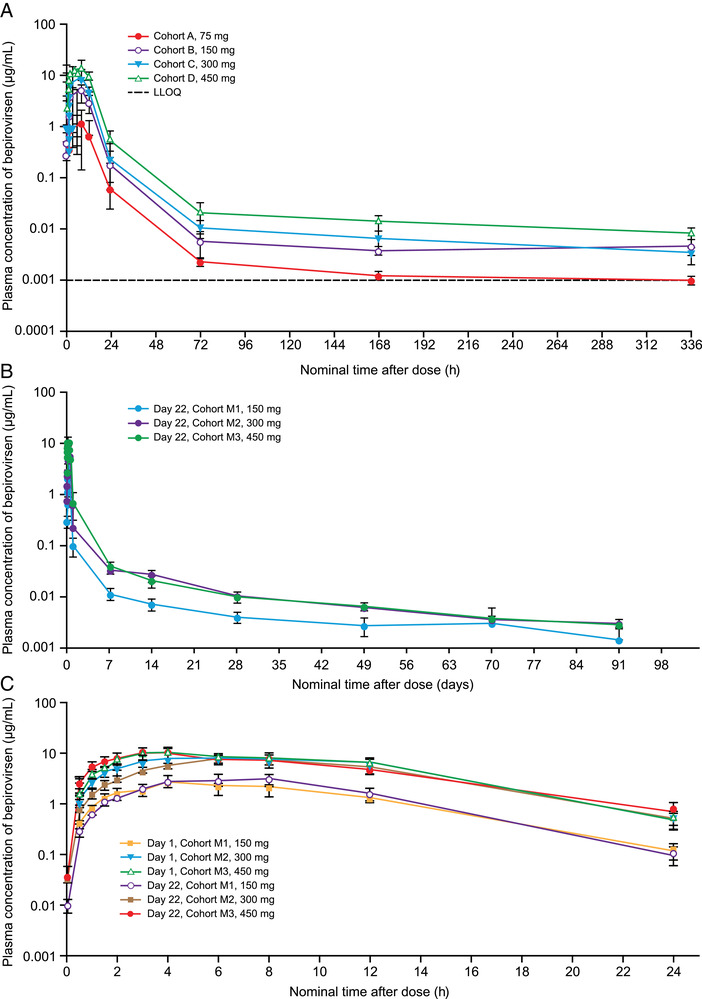
Mean (±standard error) plasma concentration of bepirovirsen in human healthy volunteers (A) single‐dose cohorts (0–336 hours), (B) multiple‐dose cohorts (0–91 days after day 22 dose), and (C) multiple‐dose cohorts on days 1 and 22. Values below the limits of quantitation were set to 0 and included in calculation of the mean. Two outliers were excluded: 4‐hour time point on day 1 for 1 participant subject (BLQ in the middle of the plasma time curve) and 8‐hour time point on day 22 for 1 participant (10‐fold lower than previous and following time point concentrations). BLQ, below LLOQ (1 ng/mL); LLOQ, lower limit of quantitation.

Following a single SC injection in all cohorts at all dose levels, the concentration‐time profile of bepirovirsen featured a rapid absorption phase, followed by an initial rapid distribution phase and a slower elimination phase (Figure 2). Median bepirovirsen t_max_ ranged from 3 to 8 hours after dosing across all cohorts and study days (Table 1). After t_max_, plasma concentrations of bepirovirsen declined in a multiexponential manner.

**Table 1:** Plasma Bepirovirsen PK Parameters in Human Healthy Volunteers (Single‐ and Multiple‐Dose Cohorts; PK Population)

Plasma Data									
Cohort (Dose)	Day	C_max_ (µg/mL)	C_168h_ ^a^ (ng/mL)	t_max_ (h)	AUC_0–24h_ (µg • h/mL)	AUC_0–168h_ (µg • h/mL)	AUC_0–336h_ (µg • h/mL)	CL/F (L/h)	t_1/2_ (days)
**A (75 mg)**	1	1.42 (0.81)	1.23 (1.06)	4 (3–6)	19.1 (6.69–31.6)^b^	21.5 (8.21–34.9)^b^	21.8 (8.45–35.2)^b^	5.50 (2.13–8.87)^b^	NA
**B (150 mg)**	1	4.67 (1.69)	3.79 (0.71)	8 (6–8)	58.3 (25.3)	63.1 (25.3)	63.8 (25.6)	2.58 (0.85)	NA
**C (300 mg)**	1	9.47 (0.34)	6.49 (2.67)	8 (6–8)	112 (5.71)	119 (6.21)	120 (6.30)	2.50 (0.13)	NA
**D (450 mg)**	1	15.8 (4.01)	14.1 (4.72)	3 (3–8)	194 (21.4)	209 (21.2)	210 (20.9)	2.15 (0.22)	NA
**Cohort M1 (150 mg)** ^d^	1	2.91 (1.24)	10.0 (5.33)^c^	4 (4–8)	31.8 (13.0)	NA	NA	NA	NA
	22	3.69 (0.40)	11.4 (4.96)	8 (4–8)	37.2 (2.88)	45.3 (7.04)	NA	3.37 (0.57)	22.4 (8.56)
**Cohort M2 (300 mg)** ^e^	1	9.25 (2.13)	34.8 (5.03)^c^	6 (4–8)	123 (3.04)	NA	NA	NA	NA
	22	7.93 (2.23)	34.4 (10.7)	6 (4–8)	103 (19.4)	148 (14.6)	NA	2.03 (0.21)	24.2 (6.06)
**Cohort M3 (450 mg)** ^f^	1	11.2 (3.89)	37.3 (10.6)^c^	3 (3–12)	139 (21.2)	NA	NA	NA	NA
	22	11.0 (4.06)	41.1 (12.0)	3 (2–4)	121 (33.3)	174 (51.4)	NA	2.75 (0.86)	24.0 (2.35)

AUC_0–24h_, area under the plasma concentration–time curve from 0 to 24 hours; AUC_0–168h_, area under the plasma concentration–time curve from 0 to 168 hours; AUC_0–336h_, area under the plasma concentration–time curve from 0 to 336 hours; CL/F, apparent clearance; C_max_, maximum concentration in plasma; NA, not applicable; PK, pharmacokinetic; SD, standard deviation; t_max_, time to C_max_; t_1/2_, terminal elimination half‐life.

n = 3 for each cohort. Data presented as arithmetic mean (SD), except t_max_, which is median (range).

^a^ Plasma concentration at 168 hours after dosing (trough concentration for weekly dosing).

^b^ Parameters could not be calculated in 1 participant due to lack of measurable drug levels in plasma after 12 hours; therefore, mean (range) is reported.

^C^ Value associated with sample collected before dosing on day 22, 168 hours after (fifth) dose administration on day 15 dose.

^d^ Multiple‐dose cohort: participants received 6 doses of 150 mg of bepirovirsen over 3 weeks (dosing on days 1, 4, 8, 11, 15, and 22).

^e^ Multiple‐dose cohort: participants received 6 doses of 300 mg of bepirovirsen over 3 weeks (dosing on days 1, 4, 8, 11, 15, and 22).

^f^ Multiple‐dose cohort: participants received 6 doses of 450 mg of bepirovirsen over 3 weeks (dosing on days 1, 4, 8, 11, 15, and 22).

Following a single SC injection of bepirovirsen, C_max_ and total exposure, expressed as the AUC, increased with increasing dose (Table 1) and appeared to be dose proportional between 150 mg and 450 mg but were lower than dose proportional between 75 mg and 150 mg (Table 1).

In the multiple‐dose cohorts, the plasma concentration–time profiles were similar to those observed following single doses (Figure 2), with a rapid initial distribution phase and a slower elimination phase. Plasma C_max_ and AUC from 0 to 24 hours were similar following the first and sixth doses (Table 1), and similar to values observed in the single‐dose cohorts at the same dose levels, suggesting little to no plasma accumulation for these parameters. C_max_ and AUC values increased with increasing dose and appeared to be dose proportional from 150 to 450 mg. Following the final dose on study day 22, the mean terminal plasma elimination half‐life was 22.4 to 24.2 days across cohorts. C_min_ concentrations of bepirovirsen measured on study day 22 were similar to those on day 29, suggesting near steady‐state plasma levels had been achieved by day 22 as a result of the loading dose schedule included in weeks 1 and 2.

Within the first 24 hours after dosing, urinary excretion of intact full‐length bepirovirsen accounted for <2% of the administered dose across all dose levels evaluated. The proportion of urinary excretion appeared to increase with increasing dose, and higher urinary excretion was observed following multiple doses. In addition, lower renal clearance was observed over the first 24 hours following a single dose than after multiple doses (Table S3).

#### Safety and Tolerability

Each participant assigned to single‐dose cohorts A, B, C, and D received 75, 150, 300, and 450 mg of bepirovirsen, respectively, on day 1; each participant in the multiple‐dose cohorts M1, M2, and M3 received cumulative doses of 900, 1800, or 2700 mg of bepirovirsen, respectively, over a span of 22–25 days.

A total of 197 treatment‐emergent AEs were reported across all dose cohorts; the treatment‐emergent AE profile was similar between single‐ and multiple‐dose cohorts. There were no serious treatment‐emergent AEs, no treatment‐emergent AEs leading to discontinuation of the study or treatment, and no deaths. Among the single‐dose cohorts, 2 participants in the placebo arm experienced 5 treatment‐emergent AEs, and 10 participants in the bepirovirsen arms experienced 36 treatment‐emergent AEs (Table S4). All but 2 (95%) of the treatment‐emergent AEs in the single‐dose cohort were mild in severity. The 2 exceptions were productive cough considered unlikely to be related to the study drug (300‐mg cohort) and face injury considered unrelated to study drug (450 mg cohort), both of which were moderate in severity. Among the multiple‐dose cohorts, 3 participants in the placebo arm experienced 13 treatment‐emergent AEs, and 9 participants in the bepirovirsen arms experienced 143 treatment‐emergent AEs (Table S5). All treatment‐emergent AEs (100%) in the multiple‐dose cohort were mild in severity and all but 2 resolved (2 reports of mild injection site erythema in 1 participant were ongoing at the end of follow‐up). Ten of 12 participants in the single‐dose cohort and 9 of 9 participants in the multiple‐dose cohort had treatment‐emergent AEs that were considered related or possibly related to bepirovirsen.

The majority of treatment‐emergent AEs (128/197; 65%) were injection‐site reactions. A subset of these, designated local cutaneous reactions at the injection site (LCRIS) and defined as treatment‐emergent AEs and presenting as pain, tenderness, erythema, pruritus or swelling at the injection site, were infrequent. One participant in the 300 mg single‐dose cohort experienced LCRIS, and all participants treated with 300 mg (n = 3) or 400 mg (n = 3) in the multiple‐dose cohorts had LCRIS following at least 1 of their 6 doses. LCRIS occurred on the day of injection and persisted for at least 2 days. All LCRIS were mild in severity, resolved spontaneously, were nonprogressive, and were not associated with any systemic sequelae.

**Figure 3:**
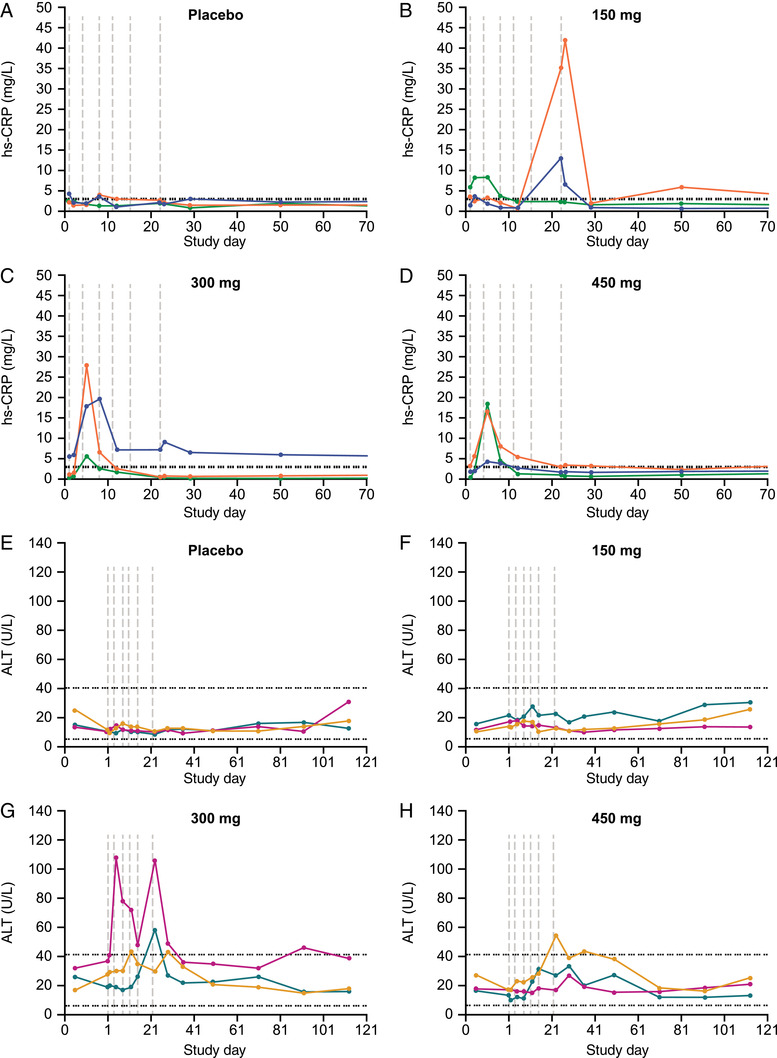
Laboratory evaluations over time per individual participant in the multiple‐dose cohorts of (A–D) hs‐CRP and (E–H) ALT (safety population). Vertical lines indicate dosing. In (A), the dotted horizontal line indicates upper limit of normal (3.0 mg/L). In (B), upper dotted horizontal line indicates upper limit of normal (41 U/L), and the lower dotted horizontal line indicates lower limit of normal (6 U/L). ALT, alanine aminotransferase; hs‐CRP, high‐sensitivity C‐reactive protein.

There were no clinically relevant changes in hematology, urinalysis, electrocardiogram, or vital signs. Six (50%) bepirovirsen‐treated participants in the single‐dose cohorts and 9 (100%) in the multiple‐dose cohorts had transient increases in C‐reactive protein (CRP) (single‐dose, Figure S11A; multiple‐dose, Figure 3A–D). Twelve participants across all cohorts had CRP increases following their first dose. Peak CRP levels ranged from 4.2 to 53.3 mg/L; those with peak CRP levels >10 mg/L (n = 5 for single‐dose; n = 6 for multiple‐dose) were reported as treatment‐emergent AEs. These increases were not associated with symptoms, and no participants had chronic CRP elevation.

Five of 21 participants dosed with bepirovirsen had ALT increases to levels greater than the upper limit of normal (ULN; 41 U/L) (single‐dose, Figure S11B; multiple‐dose, Figure 3E–H). For all but 1 of these participants (in the 300‐mg multiple‐dose group) the maximum ALT levels ranged from 43 to 58 U/L (1.03–1.41 × ULN). The participant in the 300‐mg multiple‐dose cohort had 2 ALT increases that reached 2.6 × ULN, which were reported as a mild treatment‐emergent AE with a possible relationship to bepirovirsen. In this participant, an increase in ALT to 108 U/L occurred on day 5 and substantially resolved during continued bepirovirsen dosing until a second elevation to 106 IU/L occurred on day 23; ALT level returned to near baseline levels by day 41. No ALT increases were associated with symptoms or increases in bilirubin levels, and ALT levels returned to baseline during posttreatment follow‐up. The plasma PK in this participant was similar to others in the 300‐mg multiple‐dose cohort.

No significant dose effect was observed on platelet count in either single‐ or multiple‐dose cohorts. Neutrophil counts stayed with normal range throughout the study in all dose cohorts.

## Discussion

Bepirovirsen was designed to target a highly conserved region of the HBV genome.^7^ In preclinical studies, bepirovirsen reduced intracellular HBV RNA and DNA replicative intermediates, which was reflected in the reduction of secreted HBsAg and HBeAg in HBV‐expressing cells and in a reduction in virion DNA in an HBV‐transgenic mouse model. This suggests a reduction in viral replication, consistent with reduction of key viral proteins as well as the HBV pgRNA (3.5‐kb transcript), which serves as a template for viral replication. These data indicate that effective antiviral and HBsAg lowering would be feasible at the predicted clinical dose. Evaluation of the clinical efficacy of bepirovirsen is an important avenue for future research, and it is currently being investigated in clinical trials for the treatment of patients with chronic HBV infection.^8^

In the current phase 1 first‐in‐human study, characterization of the plasma PK profile in healthy volunteers showed that bepirovirsen was absorbed rapidly into the systemic circulation, with C_max_ observed 3–8 hours after single‐ and multiple‐dose SC administration. Plasma exposure increased with increasing dose and appeared to be dose proportional at higher doses (150–450 mg) with little to no accumulation following repeat dosing. Plasma trough concentrations of bepirovirsen in the multiple‐dose groups also increased with increasing dose, reflecting tissue exposure.^16^, ^18^ Owing to a loading dose regimen, steady‐state concentrations were achieved rapidly by day 22. Renal clearance was a minor route of elimination for full‐length bepirovirsen, with <2% of the total dose eliminated in urine. Higher urinary excretion was observed following multiple doses as compared to a single dose, indicating cumulative urinary excretion of oligonucleotides associated with previous doses. The primary route of elimination is expected to be nuclease‐mediated metabolism in tissues and urinary excretion of chain‐shortened metabolites of bepirovirsen.^17^, ^19^

Members of the 2′‐MOE antisense oligonucleotide drug class have similar physicochemical properties, leading to similar PK behavior.^10^, ^14^, ^16^ The rapid decline in plasma concentration of bepirovirsen and the low level of renal elimination of full‐length drug over the first 24 hours is as expected for a compound of this chemical class and is consistent with distribution to tissues rather than elimination/excretion. This observation across the drug class along with the long half‐life of bepirovirsen supports a weekly dosing schedule.

Evaluation of safety and tolerability in this phase 1 study did not identify any safety signals that would preclude further development of the compound, with 99% of the total treatment‐emergent AEs across the single‐ and multiple‐dose cohorts being mild in severity. The majority of treatment‐emergent AEs were related to local SC reactions at the injection site (65%). Asymptomatic increases in CRP were observed in the single‐ and multiple‐dose cohorts. The data indicate that a single dose can induce CRP increases, but these are not compounded by subsequent doses of bepirovirsen. Transient CRP increases have been observed following the dosing of some but not all 2′‐MOE antisense oligonucleotides.^20^ As such, it is not clear if early CRP increases are a drug class effect but should be monitored in future studies with 2′‐MOE antisense oligonucleotides. Minor asymptomatic increases in ALT were observed in this study. One participant experienced an ALT increase to ≤2.6 × ULN without clinically relevant changes to total bilirubin and did not require dose interruption. The US Food and Drug Administration guidance on drug‐induced liver injury states that an excess of ALT increases >3 × ULN compared to a control group could be a useful indicator of the potential for liver injury. No participants in the study reached that ALT threshold. However, given the small sample size, continued assessment of liver chemistry is warranted to exclude the potential for drug‐induced liver injury. Although clinically relevant increases in ALT are not typically observed with antisense oligonucleotides,^21^ ALT increases were observed with mipomersen and ISIS 449884, probably related to their pharmacological effect, that is, inhibition of apoB100 and glucagon receptor.^22^, ^23^, ^24^

Overall, the results from this first‐in‐human study indicate that bepirovirsen, when given as single and multiple doses to healthy volunteers, showed an acceptable safety profile, with a plasma PK profile similar to other 2′‐MOE antisense oligonucleotides. The preclinical experiments supported effective antiviral activity at the doses assessed in the phase 1 study. These results support the further evaluation of bepirovirsen at doses up to 450 mg per week in participants with chronic hepatitis B.

## Conflicts of Interest

Kelong Han, Dickens Theodore, Steve Hood, and Melanie Paff are employees and stock/shareholders of GSK. T. Jesse Kwoh, C. Frank Bennett, Gina McMullen, Eric Swayze and Michael McCaleb are employees and stock/shareholders of Ionis Pharmaceuticals Inc. Gaetan Billioud and Stefan Wieland declare no conflicts of interest. Lipofectamine, PicoGreen, RiboGreen and StepOne are registered trademarks of Thermo Fisher Scientific (Waltham, Massachusetts, USA). DNeasy and RNeasy are registered trademarks of Qiagen (Hilden, Germany). WinNonlin is a registered trademark of Certara (Princeton, New Jersey, USA).

## Funding

This phase 1 first‐in‐human study was, at the time of the trial, fully sponsored and funded by Ionis Pharmaceuticals, Inc. (ISIS 50538‐CS1, NCT03020745). The study has since been acquired by GSK (Study 213725). The preclinical in vitro and in vivo studies (3470‐144) were sponsored and funded by Ionis Pharmaceuticals Inc. and supported by a grant from the National Institutes of Health to Stefan Wieland (AI094409).

## Data Sharing Statement

The data that support the findings of this analysis may be available from GSK upon request and approval from www.clinicalstudydatarequest.com. Restrictions apply to the availability of these data.

## Supporting information

Supporting Information
