# Supplementary material for: Preclinical and Phase 1 Assessment of Antisense Oligonucleotide Bepirovirsen in Hepatitis B Virus–Transgenic Mice and Healthy Human Volunteers: Support for Clinical Dose Selection and Evaluation of Safety, Tolerability, and Pharmacokinetics of Single and Multiple Doses
Source: Clin Pharmacol Drug Dev. 2022 Aug 16;11(10):1191–202. doi: 10.1002/cpdd.1154 (PMC9804925; doi:10.1002/cpdd.1154)
Supplement: Supplementary file 1 — Supporting Information [file CPDD-11-1191-s001.docx]

# SUPPLEMENTARY INFORMATION

## Supplementary Figure 1. Dose-dependent reductions in intracellular a) HBV RNA, b) HBV DNA and secreted c) HBsAg, and d) HBeAg 96 hours after bepirovirsen transfection into HepG2.2.15 cells. Results are expressed as the mean percentage of untreated control cells ± SD.


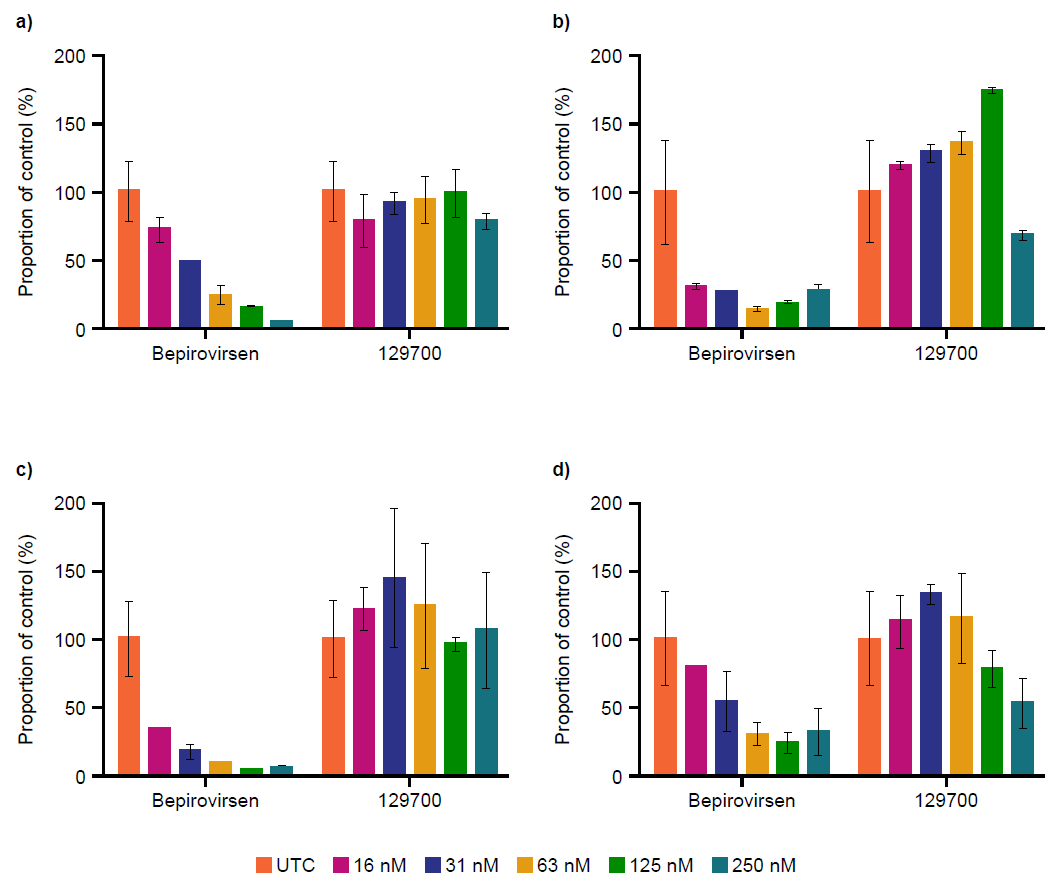


129700: control oligonucleotide (see **Supplementary Table 1**).
HBeAg, hepatitis B e antigen; HBsAg, hepatitis B surface antigen; HBV, hepatitis B virus; SD, standard deviation; UTC, untreated control.

## Supplementary Figure 2. Reduction of HBV RNA with lead HBV ASOs with multiple primer-probe sets


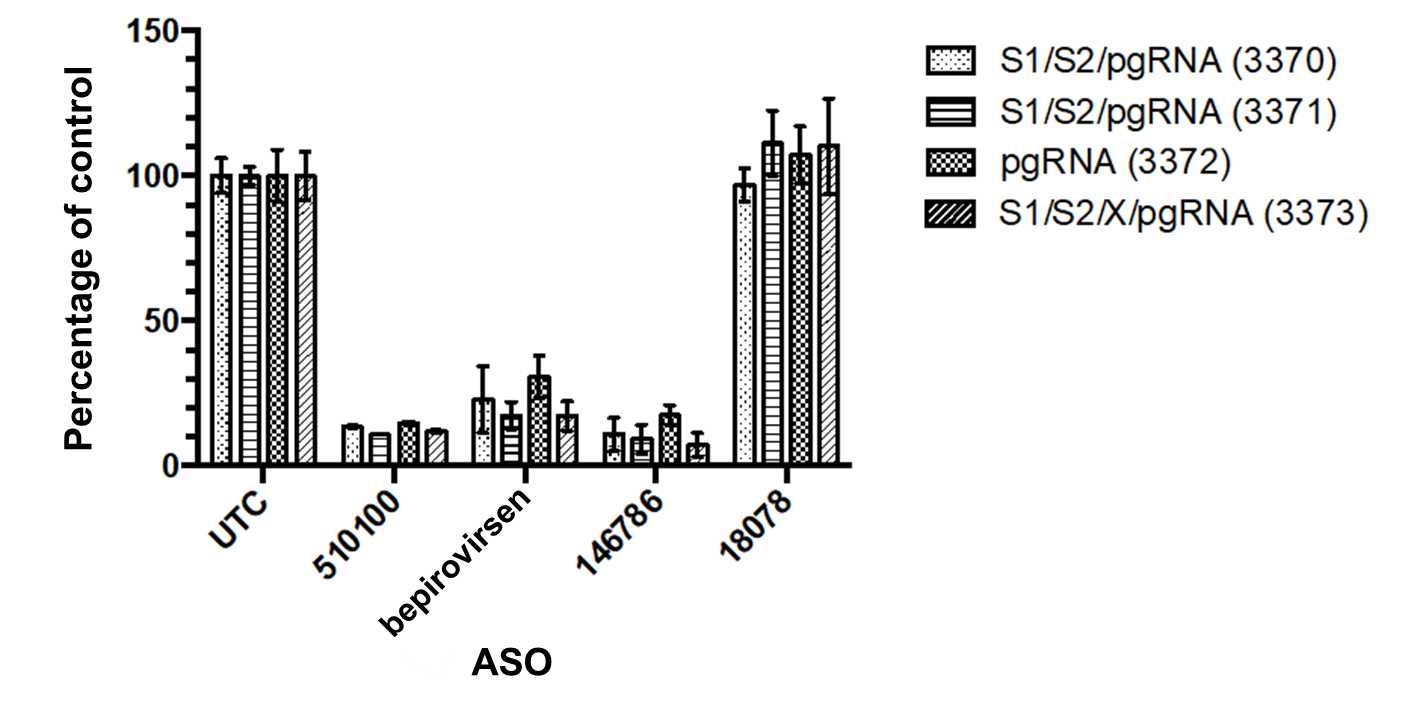


510100, bepirovirsen, 146786: HBV-targeting antisense oligonucleotides; 18078: control oligonucleotide (see **Supplementary Table 1**).
ASO, antisense oligonucleotide; HBV, hepatitis B virus; RNA, ribonucleic acid; UTC, untreated control.

## Supplementary Figure 3. Reductions in HBV DNA at 4 days following coadministration of bepirovirsen with a) ETV or b) TDF in HepG2.2.15 cells. Results are expressed as the mean percentage of untreated control cells ± SD.


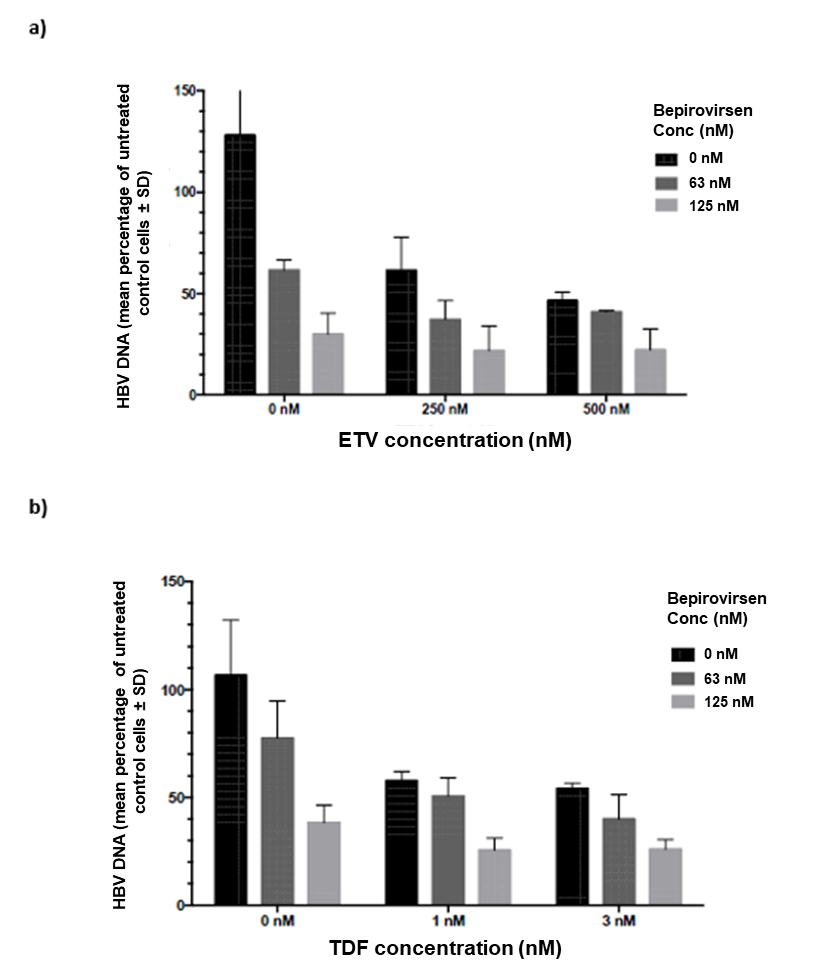


DNA, deoxribonucleic acid; ETV, entecavir; HBV, hepatitis B virus; SD, standard deviation; TDF tenofovir disoproxil fumarate.

## Supplementary Figure 4. Dose-dependent reductions in hepatic levels of a) HBV RNA and b) HBV DNA in HBV transgenic mice following treatment with vehicle of bepirovirsen at 22 or 50 mg/kg/week for 4 weeks. N=8 mice per group. Data are expressed as geometric mean ± 95% CI.


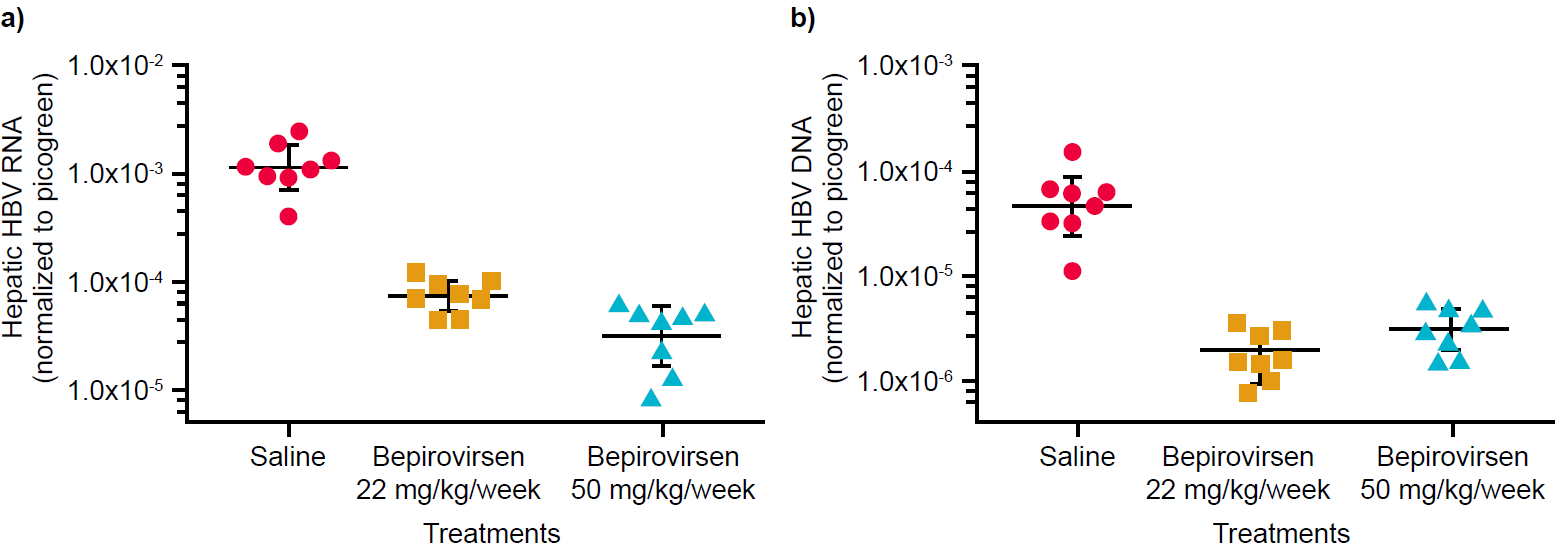


CI, confidence interval; DNA, deoxribonucleic acid; HBV, hepatitis B virus; RNA, ribonucleic acid.

## Supplementary Figure 5. Dose-dependent reduction in serum HBV DNA in HBV transgenic mice treated with saline or bepirovirsen for 4 weeks. N=8–12 mice per group. Data are expressed as geometric mean ± 95% CI.


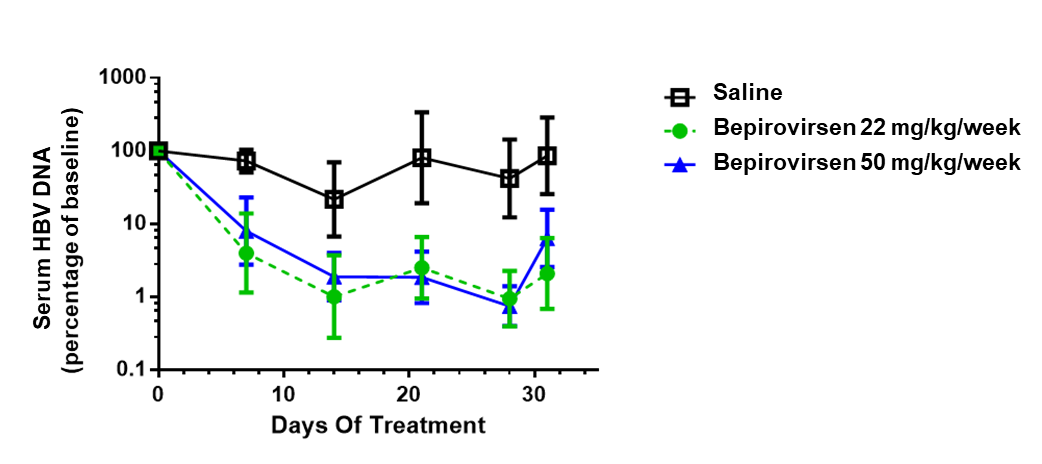


CI, confidence interval; DNA, deoxribonucleic acid; HBV, hepatitis B virus.

## Supplementary Figure 6. Dose-dependent reduction in HBsAg in HBV transgenic mice treated with saline or bepirovirsen for 4 weeks. N=8–12 mice per group. Data are expressed as arithmetic mean ± SD.


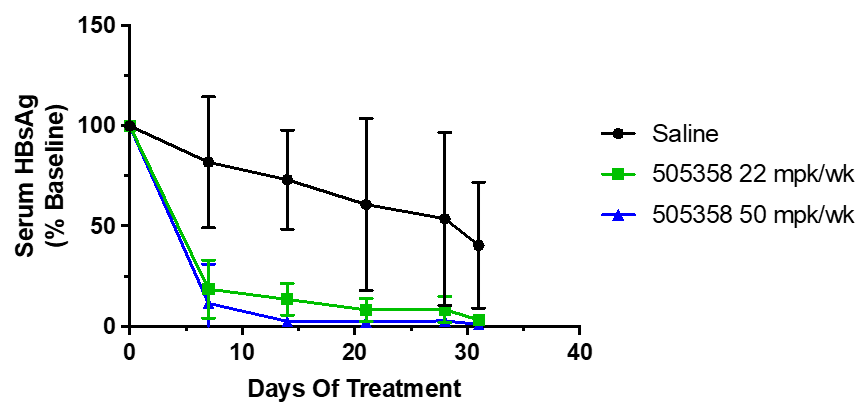


Values below LLOQ were entered as LLOQ for the calculation of mean.

HBsAg, hepatitis B surface antigen; HBV, hepatitis B virus; LLOQ, lower limit of quantification; SD, standard deviation; wk, week.

## Supplementary Figure 7. Dose-dependent reduction in serum HBeAg in HBV transgenic mice treated with saline or bepirovirsen for 4 weeks. N=8–12 mice per group. Data are expressed as a) geometric mean ± 95% CI and b) arithmetic mean ± SD.

**a)**
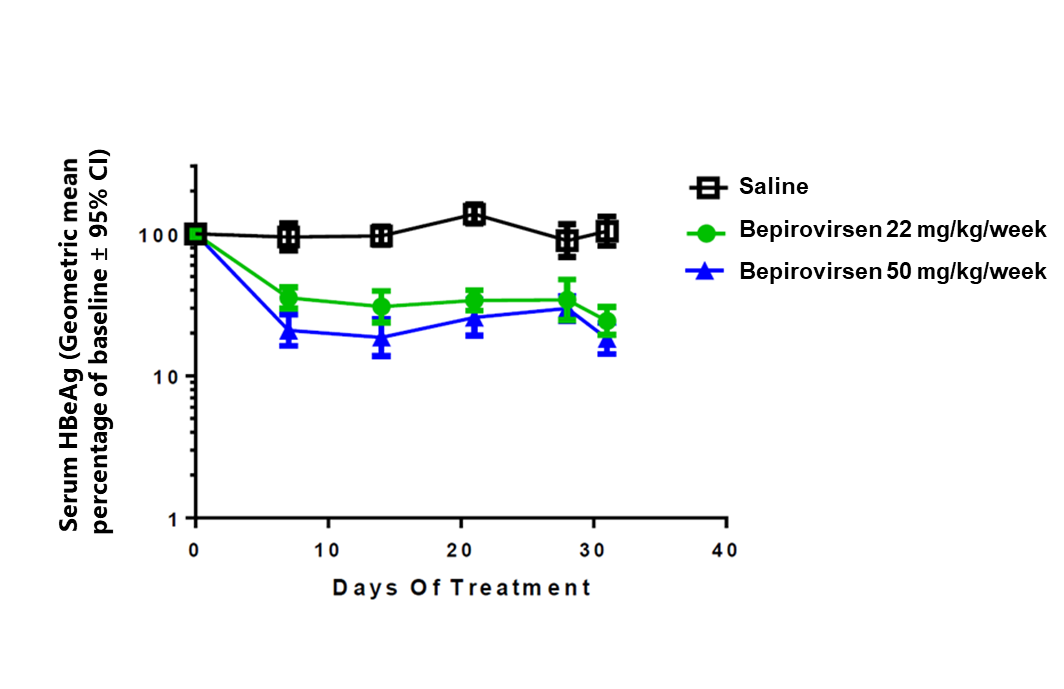


**b)**

b) Values below LLOQ were entered as LLOQ for the calculation of mean.

CI, confidence interval; HBeAg, hepatitis B e antigen; HBV, hepatitis B virus; LLOQ, lower limit of quantification; SD, standard deviation; wk, week.

## Supplementary Figure 8. HBcAg staining of liver section from representative HBV transgenic mice treated with saline or bepirovirsen for 4 weeks. The diffuse (non-focal) staining of cytoplasm by anti-HBcAg antibody was mostly diminished following bepirovirsen treatment.


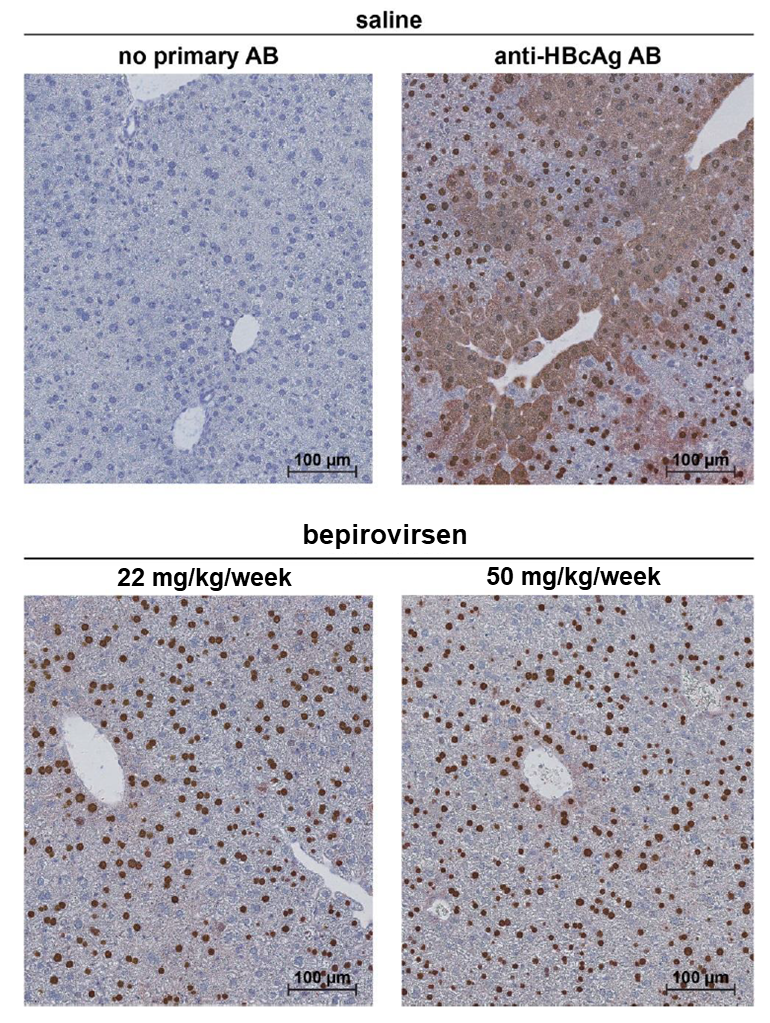


AB, antibody; HBcAg, hepatitis B core antigen; HBV, hepatitis B virus.

## Supplementary Figure 9. Dose-dependent reduction in hepatic a) HBV RNA and b) HBV DNA (using primer probe set 3371) in HBV transgenic mice treated with saline or bepirovirsen for 4 weeks alone or with co-administration of entecavir (1 mg/kg/day). N=8–12 mice per group. Data are expressed as mean percentage of saline group ± SEM.


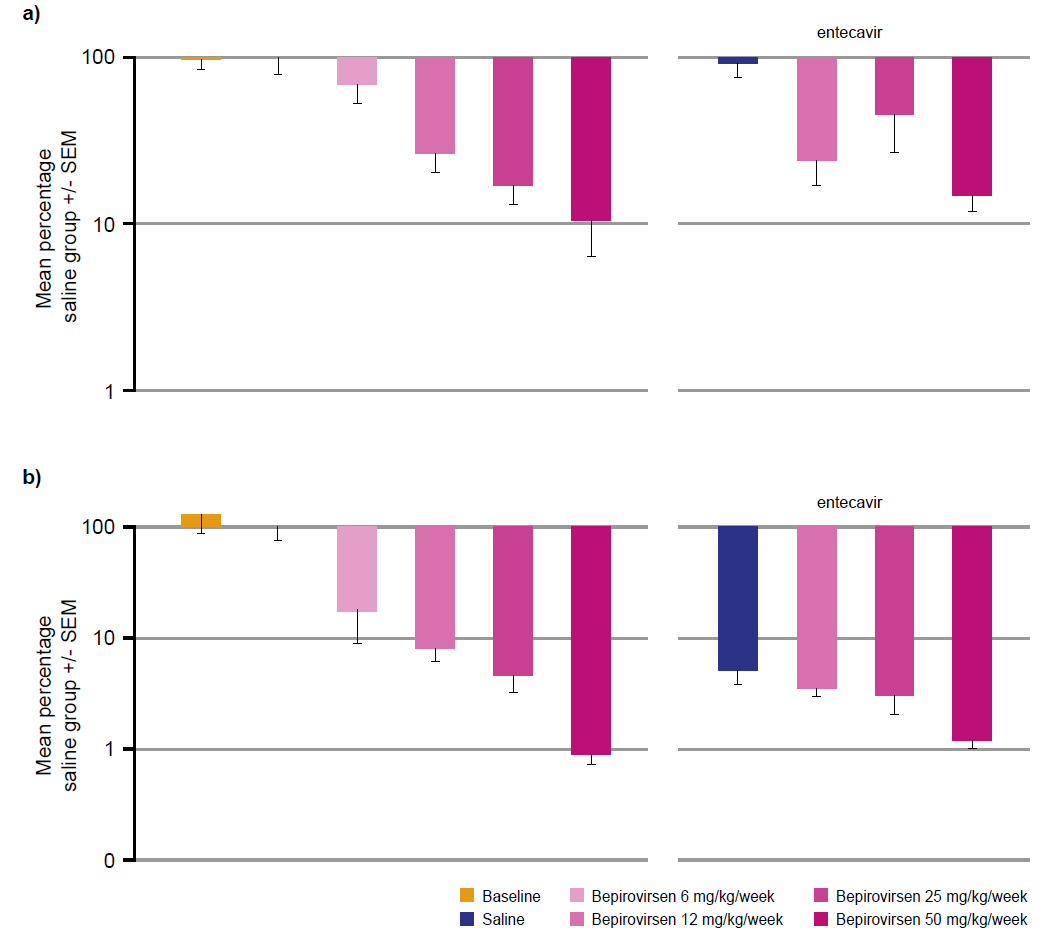


DNA, deoxyribonucleic acid; HBV, hepatitis B virus; RNA, ribonucleic acid; SEM, standard error of the mean.

## Supplementary Figure 10. Phase 1 study participant disposition.


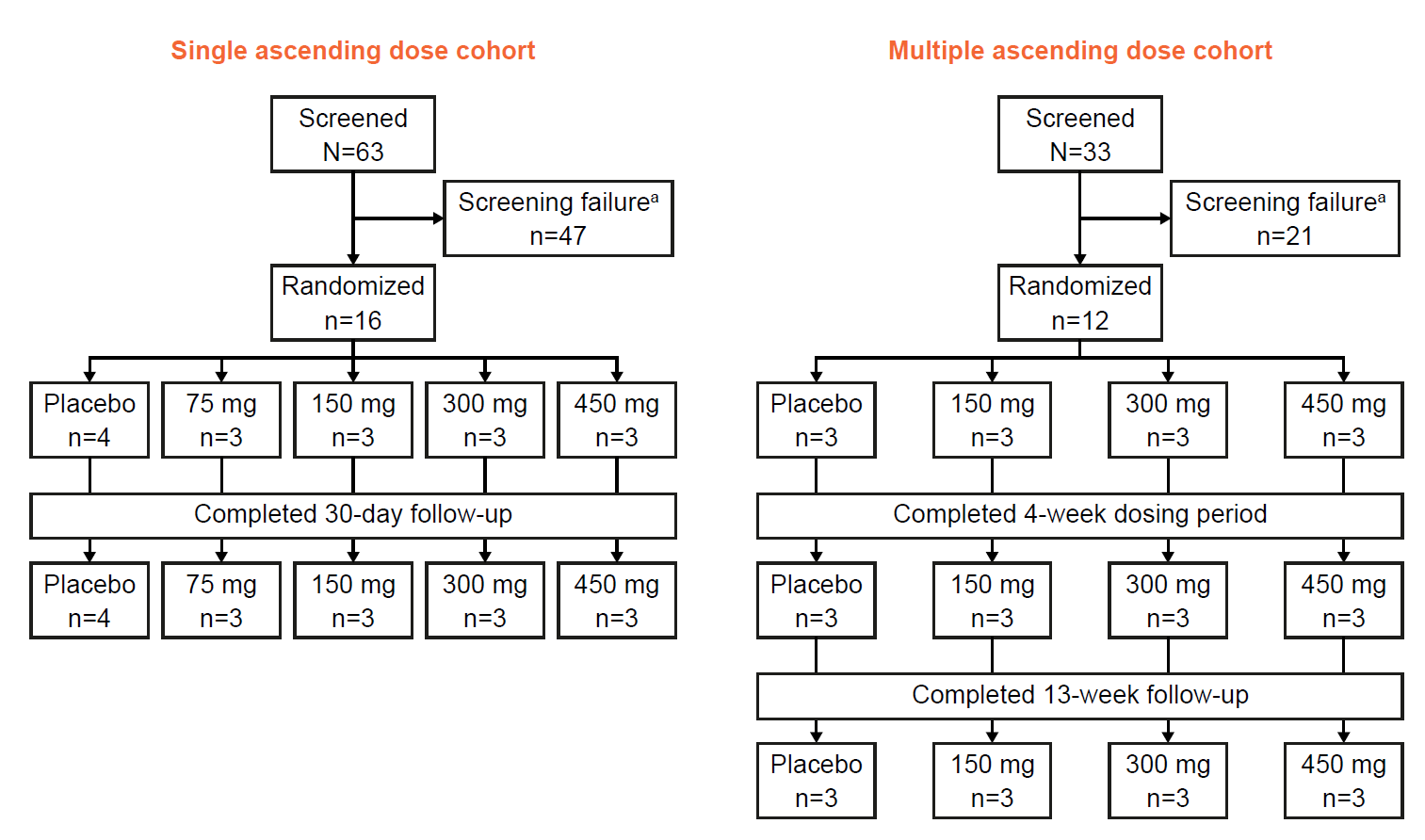


^a^Reasons for screening failure were: failure to meet inclusion/exclusion criteria (single dose, n=22; multiple dose, n=5 [abnormal laboratory results, n=15; other condition unsuitable for inclusion, n=9; positive urine drug screen, n=2; volunteer of childbearing potential, n=1]); withdrawal of consent (single dose, n=1; multiple dose, n=1); standby participant not dosed (single dose, n=4; multiple dose, n=7); other (single dose, n=20; multiple dose, n=8).

## Supplementary Figure 11. Laboratory evaluations over time per individual participant in the single-dose cohorts a) hs-CRP b) ALT (safety population).

**a)**


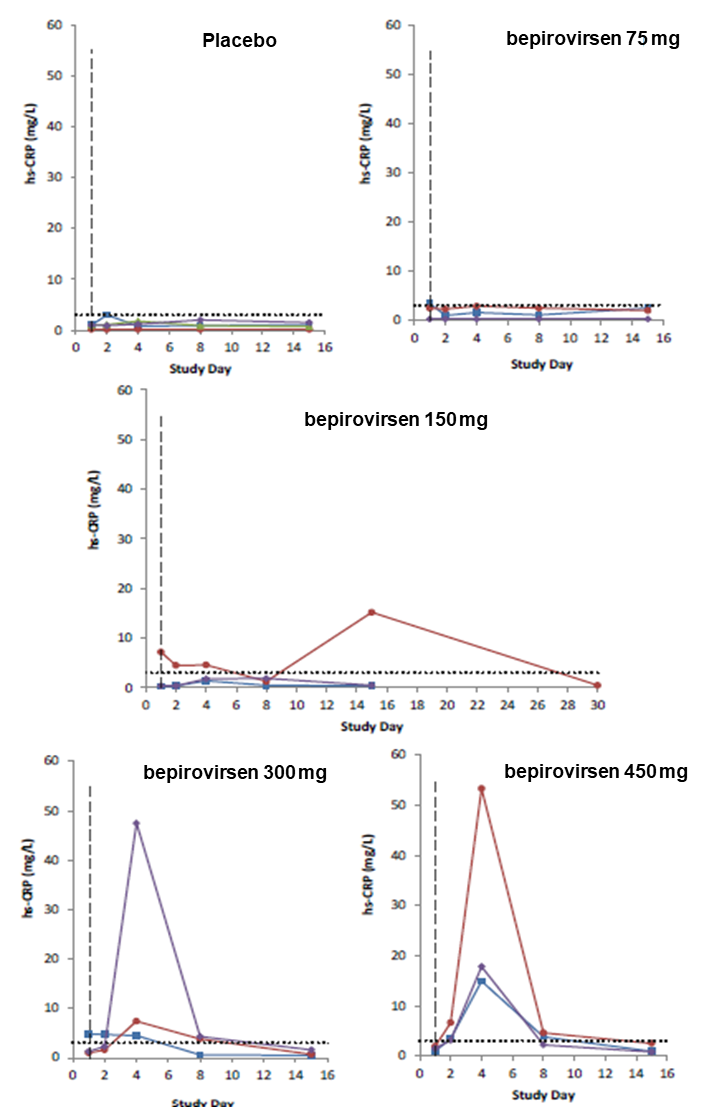


**b)**


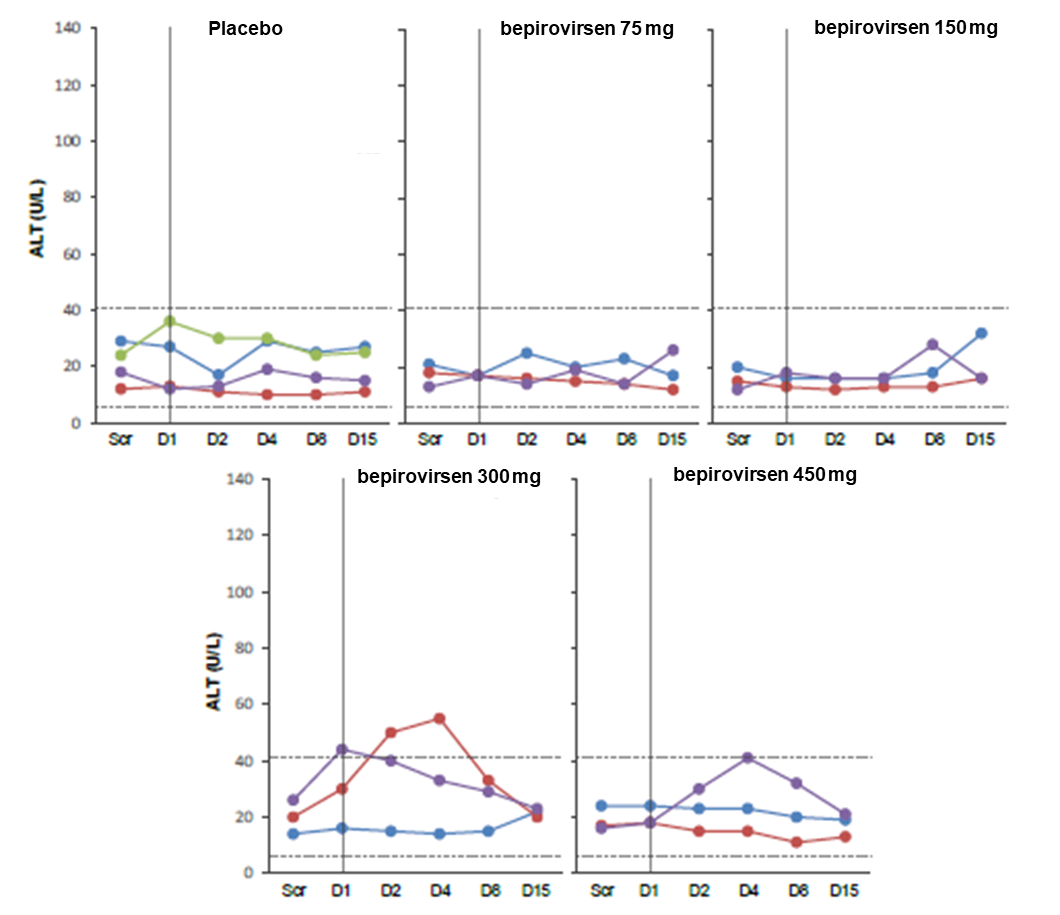


Vertical lines indicate dosing. In (a), the dotted horizontal line indicates upper limit of normal (3.0 mg/L). In (b), upper dotted horizontal line indicates upper limit of normal (41 U/L) and the lower dotted horizontal line indicates lower limit of normal (6 U/L).

ALT, alanine aminotransferase; hs-CRP, high-sensitivity C-reactive protein.

## Supplementary Table 1. Sequences, structures, and targets of ASOs.

| **Oligonucleotide** | **Sequence and Structure (5’ to 3’)^a^** | **Target** |
| --- | --- | --- |
| Bepirovirsen (505358) | G_es_^m^C_es_A_es_G_es_A_es_G_ds_G_ds_T_ds_G_ds_A_ds_A_ds_G_ds_^m^C_ds_G_ds_A_ds_A_es_G_es_T_es_G_es_^m^C_e_ | HBV |
| 129700 | T_es_A_es_G_es_T_es_G_es_^m^C_ds_G_ds_G_ds_A_ds_^m^C_ds_^m^C_ds_T_ds_A_ds_^m^C_ds_^m^C_ds_^m^C_es_A_es_^m^C_es_G_es_A_e_ | None |
| 510100 | G_es_G_es_^m^C_es_A_ds_T_ds_A_ds_G_ds_^m^C_ds_A_ds_G_ds_^m^C_ds_A_ds_G_ds_G_es_A_es_T_es_G_e_ | HBV |
| 146786 | G_es_T_es_G_es_A_es_A_es_G_ds_^m^C_ds_G_ds_A_ds_A_ds_G_ds_T_ds_G_ds_^m^C_ds_A_ds_^m^C_es_A_es_^m^C_es_G_es_G_e_ | HBV |
| 18078 | G_es_T_es_G_es_^m^C_es_G_es_C_ds_G_ds_C_ds_G_ds_A_ds_G_ds_C_ds_C_ds_C_ds_G_es_A_es_A_es_A_es_T_es_^m^C_e_ | None |

^a^Structure code is nucleotide units 5’ to 3’. Capital letters represent base codes. Small subscript are sugar and linkage codes.

ASO, antisense oligonucleotide; A, adenine; C, cytosine; _d_, deoxyribose sugar; _e_, MOE sugar; G, guanine; HBV, hepatitis B virus; ^m^C, 5-methylcytosine; MOE, 2’-O-methoxyethyl; PS, phosphorothioate; _s_, PS linkage; T, thymine.

**Supplementary Table 2. Phase 1 study baseline demographics and clinical characteristics**

|  | **Single-dose cohorts** | | | | | **Multiple-dose cohorts** | | | |
| --- | --- | --- | --- | --- | --- | --- | --- | --- | --- |
|  | **Cohort A**  **bepirovirsen  75 mg  (n=3)** | **Cohort B**  **bepirovirsen**  **150 mg (n=3)** | **Cohort C**  **bepirovirsen 300 mg (n=3)** | **Cohort D**  **bepirovirsen  450 mg  (n=3)** | **Placebo (n=4)** | **Cohort M1**  **bepirovirsen 150 mg (n=3)** | **Cohort M2**  **bepirovirsen 300 mg (n=3)** | **Cohort M3**  **bepirovirsen 450 mg (n=3)** | **Placebo (n=3)** |
| **Age (years)** |  |  |  |  |  |  |  |  |  |
| Mean (SD) | 44.3 (14.1) | 53.3 (7.6) | 45.0 (15.6) | 53.5 (5.7) | 44.8 (15.4) | 55.3 (7.4) | 54.3 (5.1) | 39.3 (11.5) | 55.3 (6.5) |
| Median (min, max) | 43 (31, 59) | 55 (45, 60) | 53 (27, 55) | 55 (47, 58) | 44 (29, 62) | 58 (47, 61) | 53 (50, 60) | 39 (28, 51) | 55 (49, 62) |
| **Sex, n (%)** |  |  |  |  |  |  |  |  |  |
| Male | 3 (100) | 2 (67) | 3 (100) | 3 (100) | 3 (75) | 3 (100) | 1 (33) | 3 (100) | 1 (33) |
| Female | 0 (0) | 1 (33) | 0 (0) | 0 (0) | 1 (25) | 0 (0) | 2 (67) | 0 (0) | 2 (67) |
| **Race, n (%)** |  |  |  |  |  |  |  |  |  |
| White | 2 (67) | 1 (33) | 3 (100) | 2 (67) | 4 (100) | 3 (100) | 3 (100) | 3 (100) | 3 (100) |
| Black or African American | 1 (33) | 2 (67) | 0 (0) | 1 (33) | 0 (0) | 0 (0) | 0 (0) | 0 (0) | 0 (0) |
| **Weight (kg)** |  |  |  |  |  |  |  |  |  |
| Mean (SD) | 90.8 (7.9) | 80.5 (16.2) | 78.8 (8.5) | 77.1 (7.9) | 75.0 (12.6) | 81.5 (9.8) | 79.4 (6.1) | 90.8 (5.8) | 69.1 (8.4) |
| Median (min, max) | 87.4 (85.2, 99.8) | 87.8 (62.0, 91.8) | 76.8 (71.5, 88.2) | 75.8 (69.9, 85.5) | 75.4 (62.8, 86.3) | 82.0 (71.5, 91.0) | 82.4 (72.4, 83.5) | 87.8 (87.1. 97.5) | 73.1 (59.4, 74.7) |
| **BMI (kg/m^2^)** |  |  |  |  |  |  |  |  |  |
| Mean (SD) | 25.9 (1.0) | 25.6 (3.3) | 27.2 (1.2) | 26.3 (3.0) | 25.2 (3.4) | 27.2 (1.4) | 28.7 (1.9) | 26.8 (0.2) | 25.5 (3.9) |
| Median (min, max) | 25.5 (25.2, 27.0) | 25.0 (22.6, 29.1) | 27.3 (26.0, 28.4) | 24.7 (24.5, 29.8) | 26.2 (20.2, 28.0) | 27.7 (25.7, 28.3) | 27.6 (27.5, 30.9) | 26.8 (26.7, 27.0) | 25.8 (21.5, 29.3) |

BMI, body mass index; SD, standard deviation.

**Supplementary Table 3. Urine bepirovirsen PK parameters in human healthy volunteers (single- and multiple-dose cohorts; PK population)**

| **Cohort (dose)** | **Day** | **Urine data** | | |  |
| --- | --- | --- | --- | --- | --- |
|  |  | **Urine conc (µg/mL)** | **Ae_24h_ (mg)** | **% of dose excreted** | **CL_r_**  **(mL/h)** |
| **A (75 mg)** | 1 | 0.05 (0.07) | 0.06 (0.07) | 0.09 (0.1) | 3.46 (1.80) |
| **B (150 mg)** | 1 | 0.07 (0.07) | 0.12 (0.05) | 0.08 (0.03) | 2.24 (1.41) |
| **C (300 mg)** | 1 | 0.26 (0.24) | 0.25 (0.13) | 0.08 (0.04) | 2.20 (1.15) |
| **D (450 mg)** | 1 | 1.27 (0.54) | 2.13 (1.16) | 0.47 (0.26) | 10.7 (5.43) |
| **M1 (150 mg)** | 1 | 0.05 (0.03) | 0.05 (0.03) | 0.03 (0.02) | 1.38 (0.69) |
|  | 22 | 2.2 (1.39) | 2.74 (0.93) | 1.83 (0.62) | 72.9 (22.3) |
| **M2 (300 mg)** | 1 | 0.45 (0.11) | 0.75 (0.15) | 0.25 (0.05) | 6.12 (1.14) |
|  | 22 | 2.75 (1.26) | 4.97 (2.94) | 1.66 (0.98) | 53.2 (41.6) |
| **M3 (450 mg)** | 1 | 1.2 (0.37) | 1.07 (0.23) | 0.24 (0.05) | 8.05 (3.09) |
|  | 22 | 3.91 (1.33) | 4.94 (3.31) | 1.10 (0.74) | 47.9 (41.5) |

n=3 for each cohort. Data presented as arithmetic mean (SD). Ae24h, amount excreted up to 24 hours; CL_r_, renal clearance; PK, pharmacokinetic.

## Supplementary Table 4. TEAEs reported by participants in the single-dose cohorts (safety population)

| **TEAE, n %**  **System Organ Class**  Preferred Term | **bepirovirsen**  **75 mg**  **(n=3)** | **bepirovirsen**  **150 mg**  **(n=3)** | **bepirovirsen**  **300 mg**  **(n=3)** | **bepirovirsen**  **450 mg**  **(n=3)** | **Total bepirovirsen**  **(n=12)** | **Placebo**  **(n=4)** |
| --- | --- | --- | --- | --- | --- | --- |
| **Participants with ≥1 TEAE** | 3 (100) | 2 (67) | 2 (67) | 3 (100) | 10 (83) | 2 (50) |
| **Gastrointestinal disorders** | 0 | 0 | 0 | 0 | 0 | 1 (25) |
| Diarrhea | 0 | 0 | 0 | 0 | 0 | 1 (25) |
| **General disorders and administration site conditions** | 2 (67) | 1 (33) |  | 2 (67) |  | 2 (50) |
| Catheter site pain | 0 | 0 | 0 | 0 | 0 | 1 (25) |
| Feeling hot | 0 | 0 | 1 (33) | 0 | 1 (8) | 0 |
| Injection site discomfort | 0 | 1 (33) | 0 | 0 | 1 (8) | 0 |
| Injection site erythema | 0 | 0 | 2 (67) | 1 (33) | 3 (25) | 0 |
| Injection site hematoma | 0 | 0 | 0 | 1 (33) | 1 (8) | 0 |
| Injection site induration | 0 | 0 | 0 | 1 (33) | 1 (8) | 0 |
| Injection site pain | 0 | 0 | 0 | 1 (33) | 1 (8) | 0 |
| Injection site pruritus | 0 | 0 | 1 (33) | 0 | 1 (8) | 0 |
| Injection site reaction^a^ | 2 (67) | 0 | 0 | 1 (33) | 3 (25) | 0 |
| Tenderness | 0 | 0 | 0 | 0 | 0 | 1 (25) |
| **Infections and infestations** | 0 | 0 | 1 (33) | 0 | 1 (8) | 0 |
| Pharyngitis | 0 | 0 | 1 (33) | 0 | 1 (8) | 0 |
| Sinusitis | 0 | 0 | 1 (33) | 0 | 1 (8) | 0 |
| **Injury, poisoning and procedural complications** | 0 | 0 | 0 | 1 (33) | 1 (8) | 0 |
| Face injury | 0 | 0 | 0 | 1 (33) | 1 (8) | 0 |
| **Investigations** | 0 | 1 (33) | 1 (33) | 3 (100) | 5 (42) | 0 |
| C-reactive protein increased | 0 | 1 (33) | 1 (33) | 3 (100) | 5 (42) | 0 |
| **Musculoskeletal and connective tissue disorders** | 0 | 0 | 1 (33) | 0 | 1 (8) | 0 |
| Myalgia | 0 | 0 | 1 (33) | 0 | 1 (8) | 0 |
| **Nervous system disorders** | 2 (67) | 0 | 1 (33) | 0 | 3 (25) | 0 |
| Headache | 1 (33) | 0 | 1 (33) | 0 | 2 (17) | 0 |
| Somnolence | 1 (33) | 0 | 0 | 0 | 1 (8) | 0 |
| **Respiratory, thoracic and mediastinal disorders** | 1 (33) | 0 | 2 (67) | 0 | 3 (25) | 0 |
| Cough | 1 (33) | 0 | 0 | 0 | 1 (8) | 0 |
| Nasal congestion | 0 | 0 | 2 (67) | 0 | 2 (17) | 0 |
| Productive cough | 0 | 0 | 1 (33) | 0 | 1 (8) | 0 |
| Sneezing | 0 | 0 | 2 (67) | 0 | 2 (17) | 0 |
| **Skin and subcutaneous tissue disorders** | 0 | 0 | 0 | 0 | 0 | 1 (25) |
| Erythema | 0 | 0 | 0 | 0 | 0 | 1 (25) |
| Pruritus | 0 | 0 | 0 | 0 | 0 | 1 (25) |

^a^Fasciculations at the injection site were coded to Injection site reaction.

TEAE, treatment-emergent adverse event.

## Supplementary Table 5. TEAEs reported by participants in the multiple-dose cohorts (safety population)

| **TEAE, n %**  **System Organ Class**  Preferred Term | **bepirovirsen**  **150 mg**  **(n=3)** | **bepirovirsen**  **300 mg**  **(n=3)** | **bepirovirsen**  **450 mg**  **(n=3)** | **Total**  **bepirovirsen**  **(n=9)** | **Placebo**  **(n=3)** |
| --- | --- | --- | --- | --- | --- |
| **Participants with ≥1 TEAE** | 3 (100) | 3 (100) | 3 (100) | 9 (100) | 3 (100) |
| **General disorders and administration site conditions** | 3 (100) | 3 (100) | 3 (100) | 9 (100) | 3 (100) |
| Chills | 1 (33) | 0 | 0 | 1 (11) | 0 |
| Fatigue | 0 | 1 (33) | 1 (33) | 2 (22) | 1 (33) |
| Injection site anesthesia | 1 (33) | 0 | 0 | 1 (11) | 0 |
| Injection site bruising | 0 | 1 (33) | 1 (33) | 2 (22) | 1 (33) |
| Injection site erythema | 0 | 3 (100) | 3 (100) | 6 (67) | 1 (33) |
| Injection site hematoma | 0 | 1 (33) | 1 (33) | 2 (22) | 0 |
| Injection site induration | 0 | 1 (33) | 0 | 1 (11) | 0 |
| Injection site edema | 0 | 0 | 2 (67) | 2 (22) | 0 |
| Injection site pain | 2 (67) | 3 (100) | 2 (67) | 7 (78) | 2 (67) |
| Injection site papule | 0 | 1 (33) | 0 | 1 (11) | 0 |
| Injection site pruritus | 0 | 2 (67) | 0 | 2 (22) | 0 |
| Injection site reaction^a^ | 3 (100) | 1 (33) | 1 (33) | 5 (56) | 0 |
| Injection site swelling | 0 | 3 (100) | 0 | 3 (33) | 0 |
| Injection site warmth | 0 | 1 (33) | 0 | 1 (11) | 0 |
| Pain | 0 | 0 | 1 (33) | 1 (11) | 0 |
| Pyrexia | 1 (33) | 0 | 0 | 1 (11) | 0 |
| Vessel puncture site hematoma | 0 | 0 | 1 (33) | 1 (11) | 0 |
| **Infections and infestations** | 1 (33) | 0 | 0 | 1 (11) | 0 |
| Oral herpes | 1 (33) | 0 | 0 | 1 (11) | 0 |
| **Investigations** | 2 (67) | 3 (100) | 2 (67) | 7 (78) | 0 |
| Alanine aminotransferase increased | 0 | 1 (33) | 0 | 1 (11) | 0 |
| Blood creatine phosphokinase increased | 0 | 1 (33) | 1 (33) | 2 (22) | 0 |
| C-reactive protein increased | 2 (67) | 2 (67) | 2 (67) | 6 (67) | 0 |
| **Musculoskeletal and connective tissue disorders** | 1 (33) | 0 | 0 | 1 (11) | 1 (33) |
| Back pain | 0 | 0 | 0 | 0 | 1 (33) |
| Myalgia | 1 (33) | 0 | 0 | 1 (11) | 0 |
| **Nervous system disorders** | 2 (67) | 0 | 2 (67) | 4 (44) | 1 (33) |
| Headache | 2 (67) | 0 | 2 (67) | 4 (44) | 1 (33) |
| Paresthesia | 1 (33) | 0 | 0 | 1 (11) | 0 |
| Somnolence | 1 (33) | 0 | 0 | 1 (11) | 0 |
| **Renal and urinary disorders** | 0 | 0 | 0 | 0 | 1 (33) |
| Dysuria | 0 | 0 | 0 | 0 | 1 (33) |
| **Respiratory, thoracic and mediastinal disorders** | 2 (67) | 0 | 0 | 2 (22) | 0 |
| Cough | 1 (33) | 0 | 0 | 1 (11) | 0 |
| Nasal congestion | 2 (67) | 0 | 0 | 2 (22) | 0 |
| Rhinorrhea | 2 (67) | 0 | 0 | 2 (22) | 0 |
| Sneezing | 1 (33) | 0 | 0 | 1 (11) | 0 |
| **Skin and subcutaneous tissue disorders** | 1 (33) | 0 | 0 | 1 (11) | 0 |
| Hyperhidrosis | 1 (33) | 0 | 0 | 1 (11) | 0 |

^a^Fasciculations at the injection site were coded to Injection site reaction.

TEAE, treatment-emergent adverse event.

## Supplementary Methods

### Preclinical studies

Histology

Mouse liver tissue was fixed in Buffered Zinc Formalin (Anatech Ltd) overnight at room temperature. Tissues were then processed and paraffin (Thermo Histoplast PE) embedded using a Thermo Excelsior processor (Thermo Fisher). Eight μm sections on glass slides were deparaffinized and rehydrated through Xylene (Pro-Par Clearant, Anatech) and a graded ethanol series. Sections were rinsed in water and wash buffer (TBST: 50mM Tris Base, 0.9% NaCl, 0.05% Tween20, pH = 8.4) for 5 min each prior to blocking for 30 min with 2.5% normal horse serum. The blocking solution was removed and replaced by TBST containing a polyclonal rabbit ant-HBcAg antibody (Dako) at a 1:100 dilution. After 45 min incubation at RT, the slides were washed twice for 5 min with TBST before quenching of endogenous peroxidase with 3% H2O2 in water for 10 min. After 2 washes (5 min each) with TBST, sections were incubated with ready to use anti-rabbit immunoglobulin solution (ImmPress Reagent, Vector Laboratories) for 30 min. After two 5 min washes in TBST, the slides were incubated with peroxidase substrate solution (Vector AEC peroxidase substrate kit, Vector labs). The peroxidase reactions were stopped by rinsing with water and slides were mounted using aqueous mounting media (Dako).

### Phase 1: clinical study

Full eligibility criteria

To be eligible, participants must have met the following eligibility criteria within 28 days prior to study Day 1 (unless otherwise specified).

Inclusion criteria

1. Have given written informed consent (signed and dated) and any authorizations required by local law and able to comply with all study requirements
2. Male or female in good health
3. Age 18 to 65 years at the time of informed consent
4. Females must be non-pregnant and non-lactating, and either surgically sterile (eg, tubal occlusion, hysterectomy, bilateral salpingectomy, bilateral oophorectomy) or postmenopausal. Males must be surgically sterile, abstinent or if engaged in sexual relations of childbearing potential, the participant must be using an acceptable contraceptive method during and for at least 30 days (single-dose cohorts) or 13 weeks (multiple-dose cohorts) after the last dose of study drug.
5. Body mass index ≤32.0 kg/m^2^

Exclusion criteria

1. Clinically significant abnormalities in medical history (eg, previous acute coronary syndrome within 6 months of screening, major surgery within 3 months of screening) or physical examination
2. Screening laboratory results as follows or any other clinically significant abnormalities in screening laboratory values that would render a subject unsuitable for inclusion
   1. Alanine aminotransferase (ALT), aspartate aminotransferase (AST), bilirubin, or alkaline phosphatase > upper limit of normal (ULN)
   2. Serum creatinine or blood urea nitrogen >ULN
   3. Platelet count < lower limit of normal
   4. Urine protein/creatinine ratio ≥0.2 mg/mg. In the event of a ratio above this threshold, eligibility may be confirmed by a quantitative total urine protein measurement of <150 mg/24 hours
   5. Trace or greater result in qualitative test for blood in urine. In the event of a positive test, eligibility may be confirmed with urine microscopy showing <5 red blood cells per high power field
   6. Positive test result for HIV, hepatitis B virus (HBV) or hepatitis C virus (HCV)
3. History of bleeding diathesis or coagulopathy
4. Active infection requiring systemic antiviral or antimicrobial therapy that will not be completed prior to Study Day 1
5. Malignancy within 5 years, except for basal or squamous cell carcinoma of the skin or carcinoma in situ of the cervix that has been successfully treated
6. Treatment with another investigational drug, biological agent, or device within 4 weeks of screening, or 5 half-lives of investigational agent, whichever is longer
7. Treatment with any oligonucleotide (not developed by Ionis Pharmaceuticals, Inc.; including small interfering RNA) at any time
8. Prior treatment with an oligonucleotide (developed by Ionis Pharmaceuticals, Inc.) within
   9 months of screening. Participants that have previously received only a single dose of an Ionis-developed oligonucleotide as part of a clinical study may be included as long as a duration ≥4 months has elapsed since dosing
9. Blood donation of 50 to 499 mL within 30 days of screening or of >499 mL within 60 days of screening
10. Regular use of alcohol within 6 months prior to screening (>7 drinks/week for females, 14 drinks/week for males (1 drink = 5 ounces [150 mL] of wine or 12 ounces [360 mL] of beer or 1.5 ounces [45 mL] of hard liquor)
11. Use of soft drugs (such as marijuana) within 3 months prior to screening, or hard drugs (such as cocaine and phencyclidine) within 1 year prior to screening, or positive urine drug screen at screening
12. Chronic or acute prescription medications unless permitted by the Medical Monitor
13. Unwillingness to comply with study procedures, including follow-up, or unwillingness to cooperate fully with the Investigator
14. Have any other conditions, which, in the opinion of the Investigator would make the subject unsuitable for inclusion, or could interfere with the subject participating in or completing the study

Stopping criteria

Stopping criteria were based on changes on liver chemistry, renal function or platelet count. If any of the following protocol-defined stopping criteria were met, the participant was permanently discontinued from treatment with bepirovirsen.

- Liver chemistry: ALT or AST >8 x ULN; ALT or AST >5 x ULN at two consecutive weekly measurements (not less than 7 days nor more than 10 days apart) both of which are confirmed. Treatment with bepirovirsen may continue until the second consecutive weekly ALT or AST measurement is confirmed to be >5 x ULN; ALT or AST >3 x ULN and total bilirubin >2 x ULN; ALT or AST >3 x ULN with the appearance or worsening of symptoms felt by the Investigator to be potentially related to hepatic inflammation such as fatigue, nausea, vomiting, right upper quadrant pain or tenderness, fever, rash, and/or concomitant eosinophilia (>5%)
- Renal function: confirmed fasting serum creatinine increase that is both ≥0.3 mg/dL (26.5 μmol/L) and ≥40% above baseline creatinine values (ie, pre-dose fasting creatinine from Study Day 1), and is >ULN; proteinuria, dipstick 2 + (confirmed by dipstick retest and then further confirmed by a quantitative total urine protein measurement of >1.0 g/24 hour)
- Platelet count: <75,000/mm^3^

Additional support for dose selection

The starting dose of 75 mg was also selected based on the no observed adverse effect level in a 13-week monkey toxicology study (12 mg/kg/week), which translated to a starting dose of <10% of 720 and 840 mg bepirovirsen in a 60 or 70 kg human, respectively, according to US Food and Drug Administration guidance. Dose selection was also supported by data from Phase 1 evaluations of other 2′-MOE ASOs,^1-8^ given the similar plasma PK profiles between compounds in this class.^9^ The starting dose of 150 mg in the multiple-dose cohorts was selected based on the tolerability of the 75 mg dose along with the predicted efficacious dose. Lastly, bepirovirsen is available at a concentration of 150 mg/mL; dose levels higher than 450 mg were not selected for study due to the number of injections required to administer >3 mL per dose.

## References

1. Ackermann EJ, Guo S, Booten S, et al. Clinical development of an antisense therapy for the treatment of transthyretin-associated polyneuropathy. *Amyloid*. Jun 2012;19 Suppl 1:43-4.

2. Geary RS, Bradley JD, Watanabe T, et al. Lack of pharmacokinetic interaction for ISIS 113715, a 2'-0-methoxyethyl modified antisense oligonucleotide targeting protein tyrosine phosphatase 1B messenger RNA, with oral antidiabetic compounds metformin, glipizide or rosiglitazone. *Clin Pharmacokinet*. 2006;45(8):789-801.

3. Graham MJ, Lee RG, Bell TA, 3rd, et al. Antisense oligonucleotide inhibition of apolipoprotein C-III reduces plasma triglycerides in rodents, nonhuman primates, and humans. *Circ Res*. May 24 2013;112(11):1479-90.

4. Jones NR, Pegues MA, McCrory MA, et al. A Selective Inhibitor of Human C-reactive Protein Translation Is Efficacious In Vitro and in C-reactive Protein Transgenic Mice and Humans. *Mol Ther Nucleic Acids*. Nov 13 2012;1(11):e52.

5. Kastelein JJ, Wedel MK, Baker BF, et al. Potent reduction of apolipoprotein B and low-density lipoprotein cholesterol by short-term administration of an antisense inhibitor of apolipoprotein B. *Circulation*. Oct 17 2006;114(16):1729-35.

6. Sewell KL, Geary RS, Baker BF, et al. Phase I trial of ISIS 104838, a 2'-methoxyethyl modified antisense oligonucleotide targeting tumor necrosis factor-alpha. *J Pharmacol Exp Ther*. Dec 2002;303(3):1334-43.

7. Kwoh TJ. An overview of the clinical safety experience of first- and second-generation antisense oligonucleotides. In: Crooke ST, ed. *Antisense Drug Technology: Principles, Strategies and Applications*. 2nd edition ed. Taylor & Francis Group; 2008:365-399.

8. Crooke ST, Baker BF, Kwoh TJ, et al. Integrated Safety Assessment of 2'-O-Methoxyethyl Chimeric Antisense Oligonucleotides in NonHuman Primates and Healthy Human Volunteers. *Mol Ther*. Oct 2016;24(10):1771-1782.

9. Geary RS, Yu RZ, Siwkowski A, Levin AA. Pharmacokinetic/pharmacodynamic properties of phosphorothioate 2ʹ-O-(2-methoxyethyl) modified antisense oligonucleotides in animals and man. In: Crooke ST, ed. *Antisense Drug Technology: Principles, Strategies and Applications*. 2nd edition ed. Taylor & Francis Group; 2008:305-326.
